# miR-17-5p Regulates Endocytic Trafficking through Targeting TBC1D2/Armus

**DOI:** 10.1371/journal.pone.0052555

**Published:** 2012-12-20

**Authors:** Andrius Serva, Bettina Knapp, Yueh-Tso Tsai, Christoph Claas, Tautvydas Lisauskas, Petr Matula, Nathalie Harder, Lars Kaderali, Karl Rohr, Holger Erfle, Roland Eils, Vania Braga, Vytaute Starkuviene

**Affiliations:** 1 BioQuant, University of Heidelberg, Heidelberg, Germany; 2 Institute for Medical Informatics and Biometry, University of Technology Dresden, Dresden, Germany; 3 Integrative Bioinformatics and Systems Biology, DKFZ, BioQuant and Institute of Pharmacy and Molecular Biotechnology, University of Heidelberg, Heidelberg, Germany; 4 Center for Biomedical Image Analysis, Faculty of Informatics, Masaryk University, Brno, Czech Republic; 5 National Heart and Lung Institute, Imperial College London, London, United Kingdom; The Ohio State University, United States of America

## Abstract

miRNA cluster miR-17-92 is known as oncomir-1 due to its potent oncogenic function. miR-17-92 is a polycistronic cluster that encodes 6 miRNAs, and can both facilitate and inhibit cell proliferation. Known targets of miRNAs encoded by this cluster are largely regulators of cell cycle progression and apoptosis. Here, we show that miRNAs encoded by this cluster and sharing the seed sequence of miR-17 exert their influence on one of the most essential cellular processes – endocytic trafficking. By mRNA expression analysis we identified that regulation of endocytic trafficking by miR-17 can potentially be achieved by targeting of a number of trafficking regulators. We have thoroughly validated TBC1D2/Armus, a GAP of Rab7 GTPase, as a novel target of miR-17. Our study reveals regulation of endocytic trafficking as a novel function of miR-17, which might act cooperatively with other functions of miR-17 and related miRNAs in health and disease.

## Introduction

MicroRNAs (miRNAs) are short non-coding RNAs (18 to 24 nt in length) regulating gene expression in metazoans. miRNAs bind to target mRNAs in a complementary or partially complementary way, resulting in degradation and/or translational repression of mRNAs [1]. miRNAs are postulated to bind to 3′ untranslated regions (3′UTRs) of transcripts [2]. Recent experimental evidence demonstrates the existence of a new class of miRNA targets containing miRNA binding sites in both their 5′UTR and 3′UTR [3], or within a coding region [4], [5]. Individual miRNA are able to simultaneously coordinate expression of numerous transcripts [6] and their encoded proteins [7], [8]. miRNAs are predicted to regulate expression of more than 60% of protein-coding mammalian genes [9] and the list of biological processes regulated by miRNAs are rapidly increasing.

Roles of miRNAs in the regulation of cell cycle progression, senescence, development and tumour biology are well established [10], [11], with numerous miRNAs identified as key regulators. One of the most studied in this context is the miRNA-17-92 cluster, called oncomir-1 and frequently over-expressed in many tumours [12]. It consists of 6 miRNAs: miR-17-5p, miR-18a-5p, miR-19a-3p, miR-20a-5p, miR-19b-3p, and miR-92a-3p (further named as miR-17, miR-18a, miR-19a, miR-20a, miR-19b and miR-92a, respectively). Series of duplications and subsequent loss of the individual members resulted in the appearance of two other paralogue clusters miR-106b-25 and miR-106a-363. Fifteen miRNAs belonging to one of these clusters can be assigned to four classes according to their seed sequences [13]. miRNAs belonging to the same class might have overlapping targets and, consequently, functions as shown by Ventura et al [14]. The importance of the miR-17-92 cluster in tumour biology is further exemplified by frequent deletion of this cluster in breast and ovarian cancers [15]. Down-regulation of members of this cluster occurs also during aging [16], haematopoietic and lung differentiation [17], [18] as well as during HIV infection [19]. Recently, miR-17-92 and both paralogue clusters were shown to be up-regulated in early re-programming stages and during induction of pluripotent stem cells [20]. Regulation of cell cycle progression accounts for the majority of these functions; and miR-17-92 was shown to either facilitate [18], [21]–[23] or inhibit cell proliferation depending on different cellular context. Individual members of the miR-17-92 cluster have been characterized to a varying degree and their functions appear to be both cooperative and individual [24]. For instance, miR-19 has been shown to be a key component in promoting *c-myc*-induced B lymphomagenesis [25], [26]. In contrast, miR-92a turned out to be dispensable in inducing lymphoma growth [12], but regulates proliferation of myeloid cells [22]. The majority of known targets of the miR-17-92 cluster have been identified for miR-17 [27], which builds miR-17 seed family with other 5 miRNAs encoded by miR-17-92 (miR-20a) and by paralogues clusters miR-106b-25 (miR-106b-5p and miR-93-5p (further called miR-106b and miR-93)) and miR-106a-363 (miR-106a-5p and miR-20b-5p (further called miR-106a and miR-20b)) [13]. miR-17 and related miRNAs target numerous regulators of cell cycle, apoptosis and transcription and, as a result, act as tumor suppressors or oncogenes [28], regulators of hematopoietic [17] and cardiopulmonary [29] systems.

Here, we show by quantitative fluorescence microscopy-based cellular assays that miRNAs sharing the miR-17 seed sequence have a broad influence on endocytic trafficking. By gene expression profiling and bioinformatics we could identify several trafficking-related genes (TBC1D2/Armus, LDLR, M6PR and ASAP2), that were down-regulated when miR-17 was over-expressed. Furthermore, by combining different approaches we validated TBC1D2/Armus, a GAP of Rab7 GTPase, as a novel target of miR-17. Our work expands the repertoire of miR-17 cellular activities beyond regulation of cell cycle and transcription. By regulating endocytic trafficking, miR-17 seed family potentially influences signalling and cell adhesion that, in turn, might cooperate with other functions of miR-17 in health and disease.

## Materials and Methods

### Materials

Polyclonal rabbit anti-TBC1D2 antibody were described in [30]. Polyclonal rabbit anti-LDLR antibody was from Cayman Chemicals (Ann Arbor, MI, USA), monoclonal mouse anti-tubulin-α (clone DM1A) was from Thermo Fisher Scientific (Waltham MA, USA), polyclonal anti-FLAG antibodies were from Sigma-Aldrich (St. Louis, USA) Secondary anti-mouse and anti-rabbit IgG HRP-conjugated antibodies were from R&D Systems (Minneapolis, MN, USA). Secondary goat Alexa647-conjugated anti-mouse IgG antibody, Dil-LDL, EGF-Alexa555 and transferrin-Alexa568 were from Invitrogen (Carlsbad, CA, USA). siRNAs targeting human TBC1D2 (SI02807518 and SI04239494), LDLR (SI00011186 and SI03024525), EGFR (SI02663983 and SI02660140), TfR (SI02780715 and SI02781121) and non-silencing siRNA “All Stars” (SI03650318) were from Qiagen (Hilden, Germany). Cy3-labeled siRNA targeting INCENP (28431) and Pre-miRs™ were from Ambion (Austin, TX, USA); miR-17-5p (PM12412), miR-18a-5p (PM12973), miR-19a-3p (PM10649), miR-20a-5p (PM10057), miR-20b-5p (PM10975), miR-92a-3p (PM10916), miR-93-5p (PM10951), miR-320a (PM11621), and Pre-miR negative control (PNC, AM17110). miR-17-5p inhibitor (Anti-miR-17-5p, AM12412) and miRNA inhibitor negative control (ANC AM17010) were from Ambion (Austin, TX, USA). TaqMan microRNA assays (ID 002308 for miR-17-5p, ID 000580 for miR-20a-5p, ID 000430 for miR-92a-3p, ID 001090 for miR-93-5p, ID 002277 for miR-320a, and ID 001093 for small nuclear RNU6B) and TaqMan gene expression assays (ID Hs00917985_m1 for TBC1D2, ID Hs00181192_m1 for LDLR, and ID Hs99999905_m1 for endogenous control GAPDH gene) were from Applied Biosystems (Foster City, CA, USA).

### Cloning of Luciferase Reporters and Plasmid Encoding LDLR

Double stranded DNA fragments encoding miRNA-binding sites and full-length 3′UTRs of TBC1D2 and LDLR genes were inserted into *Xho*I- and *Not*I-digested psiCheck-2 vector (Promega, Madison, WI, USA) immediately downstream of the stop codon of the *Renilla* luciferase gene. The plasmids with mutated or deleted miR-17-5p binding site in the TBC1D2 3′UTR were generated by using the Phusion site-directed mutagenesis kit (Finnzymes, Vantaa, Finland). HeLa cells were co-transfected with the respective luciferase reporter construct, Pre-miRs and controls at a final concentration of 50 nM. *Renilla* and firefly luciferase activities were measured consecutively using the Dual-Luciferase Assay System (Promega, Madison, WI, USA) 48 h after transfection following the manufacturer’s protocol on Glomax 96-microplate luminometer (Promega, Madison, WI, USA). cDNA of LDLR was reverse-transcribed from total RNA of HeLa cells, cloned into *Sac*I- and *Not*I-digested pEYFP-N1 vector (Clontech Laboratories, Inc., Mountain View, CA, USA). EYFP was removed by the endonucleases mentioned, and the identity of the clone confirmed by DNA sequencing. TBC1D2/Armus_547–928_ was as described in [30].

### Transfection with Pre-miRs, siRNAs and cDNAs

HeLa cells (ATCC Catalog No. CCL-2) were maintained in DMEM supplemented with 10% FCS, 100 units/ml penicillin, 100 µg/ml streptomycin, and 2 mM L-glutamine. 6×10^3^–1, 8×10^4^ cells/well and 2×10^4^–4×10^4^ cells/well were seeded on 8-well μ-slides (Ibidi, Martinsried, Germany) and 24-well plates (Greiner Bio-One, Kremsmuenster, Austria) 24 h before the transfection, respectively. We have used Lipofectamine^TM^2000 (Invitrogen, Carlsbad, CA, USA) according producers recommendations, siRNAs, Pre-, Anti-miRs at a final concentration of 50 nM and cDNAs at a final concentration of 0.65 ng/µl, respectively.

### miRNA and mRNA qRT-PCR

For miRNA qRT-PCR, HeLa cells were grown in 24-well plates and lysed 24 h, 48 h and 72 h after transfection with Pre-miRs and the respective controls. Total RNA was isolated using *mir*Vana™ miRNA Isolation Kit (Ambion, Austin, TX, USA) according to the manufacturer’s recommendations. 5 ng of total RNA was used for quantification of miRNA expression. qRT-PCR was carried out in 7500 Real-Time PCR system (Applied Biosystems, Foster City, CA, USA). The 2^−ΔΔCT^ method for the data quantification was applied [31], and miRNA expression levels were calculated relative to small nuclear RNU6B RNA. The qRT-PCR of TBC1D2 and LDLR mRNAs was performed by using 65 ng of cDNAs with the respective TaqMan mRNA Assays and carried out in StepOnePlus Real-Time PCR system (Applied Biosystems, Foster City, CA, USA). Relative TBC1D2 and LDLR expression was calculated by 2^ΔΔCT^ method using expression level of GAPDH as a reference for the quantification. Every qRT-PCR experiment for both miRNAs and mRNAs was repeated 2–3 times starting from cell seeding and transfection, and 2 replicates of reverse transcription reaction and 2 replicates of real-time PCR were run for every RNA specimen.

### Western Blot Analysis

HeLa cells were transfected with Pre-miR-17-5p, siRNAs against TBC1D2 and LDLR, and appropriate negative controls in 24-well plates. Cells for TBC1D2 analysis were lysed in 30 µl of pre-heated (95°C) lysis buffer supplemented with 10 mM DTT and proteins separated on 8% SDS-PAGE gels. Cells for LDLR was lysed in 100 µl lysis buffer (2,9% glycerol, 0,66 mM DTT, 1% SDS, 20,8 mM Tris-HCl, pH 6,8, 1 mM PMSF, 0,0025% Bromophenol Blue) and 20 µl of lysates were separated on 7% SDS-PAGE gels. Before electrophoresis, lysates were treated with benzonase nuclease (Sigma-Aldrich, St. Louis, MS, USA). Proteins were blotted on PVDF Immobilon-P membranes (Millipore, Billerica, MA, USA) and non-specific binding was blocked with 5% non-fat milk in TBS. Blots were probed with the primary antibodies in TBS-0,1% Tween-20 for 2 h at RT and with the secondary HRP-conjugated antibodies in TBS-0,1% Tween-20. Proteins were detected using ECL reagents (GE Healthcare, Little Chalfont, UK). Images were acquired using ChemoCam Imager (Intas, Ahmedabad, India) and quantified using ImageJ software (NIH, Bethesda, MD, USA).

### Dil-LDL, EGF-Alexa555 and Transferrin-Alexa568 Internalization Assays

HeLa cells were plated at a density of 8×10^3^ on 8-well μ-slides (Ibidi, Martinsried, Germany), cultured for 24 h and transfected with Pre-miRs, siRNAs and appropriate negative controls. Transfected cells were incubated in a full growth medium for 24 h. For Dil-LDL internalisation assay, medium was exchanged for DMEM supplemented with 2 mM L-glutamine and 0,2% (w/v) BSA. 48 h after transfection 2-hydroxy-β-cyclodextrin (HPCD) (Sigma-Aldrich, St. Louis, MS, USA) was added at a final concentration of 10 mg/ml for 45 min. Dil-LDL cellular uptake assay was performed as described elsewhere [32]. Prior to EGF-Alexa555 uptake assay, cells were starved for 12 h. EGF-Alexa555 internalization assay was performed as described elsewhere [33]. Prior to transferrin-Alexa568 uptake assay, cells were maintained in the starvation medium supplemented with 1 mg/ml BSA for 1 h and transferrin-Alexa568 internalization assay was performed as described previously [34]. Then cells were fixed in 3% paraformaldehyde, nuclei stained with 0,3 µg/ml Hoechst 33342, and images were acquired on Sca
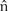
R (Olympus, Tokyo, Japan) automated microscope using 10× UplanSApo objective (NA 0.4). Sca
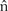
R software (version: 2.1.0.16) was used to acquire images. To image Dil-LDL internalisation, two excitation/emission channels were used: excitation wavelength = 325–375 nm, emission wavelength = 435–475 nm was used to image nuclei, excitation wavelength = 426–446 nm, emission wavelength = 460–500 nm - to image Dil-LDL and EGF-Alexa-555. To image internalized transferrin-Alexa568, 545–580 nm excitation and 610–700 emission wavelengths were used.

### Automated Classification of Nuclei

HeLa cells were transfected on 8-well μ-slides and incubated for 24–72 h. Before imaging, cell nuclei were stained with 0,3 µg/ml Hoechst 33342 and images were acquired using Sca
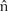
R (Olympus, Tokyo, Japan) with 20× UplanSApo (NA 0.75) and 40× UplanSApo (NA 0.9) objectives at 37°C in the presence of 5% CO_2_. Segmentation of nuclei, grey value normalization across the experiments, feature extraction and classification into four defined classes was applied sequentially to analyse the images automatically. A gradient based thresholding approach for the segmentation of cell nuclei, feature extraction and object classification was applied as described earlier [35], [36]. For training of the classifier, a set of 577 images from six independent experiments were manually annotated. For each experiment more than 3500 nuclei were analysed.

### mRNA Expression Analysis

Total RNA was isolated from HeLa cell using *mirVana*™ miRNA Isolation Kit (Ambion, Austin, TX, USA) 12 h, 24 h and 48 h after transfection with Pre-miR-17-5p and controls. The quality of RNA was checked by gel analysis on an Agilent 2100 Bioanalyzer (Agilent Technologies, Santa Clara, CA, USA) and estimated by calculating the 28S/18S ratio of ribosomal RNAs by RIN algorithm [37]. 250 ng of total RNA were used for complementary DNA (cDNA) synthesis followed by the amplification and labelling step to synthesize biotin-labeled cRNA with MessageAmp II aRNA Amplification kit (Ambion, Austin, TX, USA). cRNA was column purified with TotalPrep RNA Amplification Kit (Ambion, Austin, TX, USA). Biotin-labeled cRNA samples were hybridized on Human Sentrix-8 BeadChip® arrays (Illumina, San Diego, CA, USA) at 58°C, in GEX-HCB buffer (Illumina, San Diego, CA, USA) at a concentration of 100 ng cRNA/µl for 20 h. The arrays were scanned on Beadstation array scanner (Illumina, San Diego, CA, USA).

### Bioinformatics Analysis

The miR-17-5p seed-binding site 5′-GCACUUU-3′ in hit mRNAs was identified by BLAST (Human genomic+transcript database, expected threshold –10, word size –28, megaBLAST). In case of several mRNA variants assigned to the same gene, all of them were analyzed. No mismatches within 5′-CACUUU-3′ sequence were allowed. Functional classification of the down-regulated transcripts was done according UniProtKB annotations (http://www.uniprot.org/uniprot/).

### Statistical Analysis

In order to quantify the uptake efficiency of a given ligand,49 images/well were acquired in 8-well µ-slides in internalisation assays. Prior to quantification of the internalised ligand-specific fluorescence intensity, background signal was subtracted applying a rolling ball algorithm implemented in the Sca
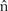
R Analysis module (Olympus). The median of internalisation rates for each sample was calculated over all cells in the corresponding images. R (http://cran.r-project.org/) and the ‘RNAither’ [38] package from Bioconductor (http://www.bioconductor.org/) were used for normalization of experimental replicates. For comparison of different experiments, the median of the negative controls was subtracted from each measurement, and the variance was adjusted by dividing by the standard deviation of the negative controls. Consequently, a positive value indicates facilitation of internalisation and a negative value indicates inhibition of internalisation. As the thresholds to identify effectors in Dil-LDL and EGF-Alexa555 assays, half of the normalised internalisation value (+/−1 for Dil-LDL and +/−2 for EGF-Alexa555) of the respective positive control was used. Since fast recycling of transferrin receptor leads to transferrin specific signal on the plasma membrane (PM), the threshold of +/−2, less than a half of the normalized transferrin uptake value with siRNA targeting TfR, was applied to identify effectors in transferrin endocytosis assay. On average, 8700, 5400 and 6400 cells were analysed in each experiment in LDL, EGF and transferrin endocytosis assays, respectively.

For the rescue experiment individual cells were divided into sub-populations according fluorescence intensity value of the over-expressed clones. Then we calculated mean value of intracellular EGF fluorescence intensity for each sub-population. Over-expression level of ECFP did not have any significant influence on intracellular EGF amount, whereas, the effect of over-expressed TBC1D2/Armus_547–928_ was concentration dependent. To quantify the rescue intracellular EGF amount in cells expressing high amounts of TBC1D2/Armus_547–928_ were normalised to intracellular EGF amount in all cells expressing ECFP. On average 7400 cells expressing ECFP and 150 cells expressing high levels of TBC1D2/Armus_547–928_ were analysed.

For microarray data analysis, each experiment was carried out in duplicates. Outliers with median absolute deviation of >2,5 were removed. Grey values of all remaining data points for a given probe were averaged (>25 probes per transcript per array) and normalized using quantile normalization. Fold-change (in a linear range) of the respective transcript was defined as the ratio of mean intensities of the respective probes derived from samples transfected with Pre-miR-17-5p to that of negative controls. A standard deviation of array was calculated as the difference of normalized expression values of transcript in the test and control samples, divided by the sum of the standard deviation of test and control samples between the replicates.

The statistical significance of changes in ligand internalization assays and protein expression levels was evaluated with two-sided, one-sample Student’s t-test. Differences of *p*≤0.05 were considered statistical significant.

## Results

### mRNA Profiling Identifies New Potential Targets of miR-17

In order to understand cellular roles of miR-17 in a more comprehensive manner we performed mRNA expression profiling in HeLa cells with miR-17 over-expressed for 12 h, 24 h and 48 h. We have chosen different time points to account for potentially different responses of mRNA:miRNA interaction in time and/or varying stability of the transcripts. Efficiency of miR-17 over-expression was tested by qRT-PCR and dual luciferase reporter assays after the longest incubation period, and it was found that up to 30-fold over-expression of miR-17 has been achieved (**Fig. S1**). Changes in mRNAs expression following miR-17 over-expression were considered significant if the difference to the negative control was more than 1.5-fold (see **Methods**). Expression of 41 mRNAs was altered after 12 h, 61 mRNAs – after 24 h and 69 mRNAs –48 h after the over-expression of miR-17 (**Table 1** summarises the results with mRNA expression threshold of 1.8 fold, the rest of the data are shown in **Table S1**). After subtracting overlapping transcripts, whose expression was changed at different time points, 138 mRNAs with altered expression levels were identified in this experiment. Of these transcripts, 60% were down-regulated upon the over-expression of miR-17 (82 transcripts). Seed matched sites were found in 63% of the down-regulated transcripts (51 transcripts), the majority of them residing in the 3′UTR (**Table S1**). Furthermore, five out of 21 known targets of miR-17 and related miRNAs sharing the same seed sequence [27] have been identified in our experiment. For instance, one of the validated targets of miR-20a is TGF-μ receptor type-2 (TGFBR2) [39], which is strongly down-regulated when miR-17 is over-expressed (**Table 1**). In agreement to published data, G1/S-specific cyclin-D1 CCND1 [40], cyclin-dependent kinase inhibitor p21/CDKN1A [41], tumour susceptibility gene 101 protein TSG101 [28] and amyloid μ precursor APP [42] have also been identified. However, this subset was among mRNAs, whose expression level changed not more than 1.3-fold (data not shown). Some of the known miR-17 targets (*e.g*., AML1, nuclear receptor coactivator (NCOA3)), validated because of changes in the protein expression level, rather than changes in mRNAs expression [17], [19], [43], were not identified in our experiment.

**Table 1:** mRNAs with the most pronounced changes of their expression level following miR-17 over-expression.

						Number of seed regions			
Time	Number	Gene symbol^a^	Gene accession number^b^	Gene name^c^	Fold-change^d^	5'UTR	CDS	3'UTR	Computational prediction^e^
12 h	1	TGFBR2	NM_001024847	Transforming growth factor, beta receptor II	−2,0			3	YES
	2	DAZAP2	NM_014764	DAZ associated protein 2	−1,9			1	YES
	3	MICA	NM_000247	MHC class I polypeptide-related sequence A	−1,8	1		1	YES
	4	TBC1D2	NM_018421	TBC1 domain family, member 2	−1,8			1	YES
24 h	1	IL6	NM_000600	Interleukin 6 (interferon, beta 2)	−2,1				NO
	2	JAK1	NM_002227	Janus kinase 1	−2,0		2	2	YES
	3	RNH1	NM_203385	Ribonuclease/angiogenin inhibitor 1	−1,9			2	YES
	4	HDHD1A	NM_012080	Haloacid dehalogenase-like hydrolase domain containing 1A	−1,9			3	NO
	5	C9orf152	NM_001012993	Chromosome 9 open reading frame 152	−1,9				NO
	6	DAZAP2	NM_014764	DAZ associated protein 2	−1,9			1	YES
	7	TGFBR2	NM_001024847	Transforming growth factor, beta receptor II	−1,9			3	YES
	8	TBC1D2	NM_018421	TBC1 domain family, member 2	−1,9			1	YES
	9	MT2A	NM_005953	Metallothionein 2A	−1,8				NO
48 h	1	IL6	NM_000600	Interleukin 6 (interferon, beta 2)	−3,4				NO
	2	KRT17	NM_000422	Keratin 17	−2,1				NO
	3	C1QTNF1	NM_198594	C1q and tumor necrosis factor related protein 1	−2,0	1			NO
	4	C8orf4	NM_020130	Chromosome 8 open reading frame 4	−2,0			1	NO
	5	CYR61	NM_001554	Cysteine-rich, angiogenic inducer, 61	−1,9				NO
	6	S100P	NM_005980	S100 calcium binding protein P	−1,9				NO
	7	CCL20	NM_004591	Chemokine (C-C motif) ligand 20	−1,9				NO
	8	IRAK2	NM_001570	Interleukin-1 receptor-associated kinase 2	−1,8			1	NO
	9	SLC16A6	NM_004694	Solute carrier family 16, member 6	−1,8			2	NO
	10	HSF2BP	NM_007031	Heat shock transcription factor 2 binding protein	−1,8		1		NO
	11	ALDH1A3	NM_000693	Aldehyde dehydrogenase 1 family, member A3	1,8			1	NO
	12	CLCA2	NM_006536	Chloride channel, calcium activated, family member 2	1,9			1	NO
	13	SLC1A3	NM_004172	Solute carrier family 1, member 3	1,9		1	2	NO
	14	OLFML1	NM_198474	Olfactomedin-like 1	1,9		1		NO
	15	KRTHB1	NM_002281	Keratin 81	1,9			1	NO
	16	KRT86	NM_002284	Keratin 86	1,9				NO
	17	ACTA2	NM_001613	Actin, alpha 2	2,1	1		1	NO
	18	TAC3	NM_001006667	Tachykinin 3	3,7				NO

a Official gene symbol in NCBI database.

b GeneBank gene accession number.

c Official gene name in NCBI database.

d Change of mRNA expression level in a linear range.

e Computational gene prediction as miR-17 targets by miRanda, Diana-microT and TargetScanHuman.

We next grouped the down-regulated transcripts into functional classes according to UniProtKB annotations (http://www.uniprot.org/uniprot/) (**Fig. S2**). 82 transcripts were categorized into twelve classes. Proteins encoded by these transcripts and regulating already known functions of miR-17, like signalling (e.g., tyrosine protein kinase JAK1), apoptosis (e.g., apoptosis inhibitor BIRC3) and cell cycle (e.g., kinesin KIF23) are making up to 25% of the potential targets. Six out of 82 potential targets regulate diverse steps of nucleic acids metabolism, like ribonuclease H1 RNH1, which degrades RNA in RNA-DNA duplexes, or tRNA guanine-N(7)-)-methyltransferase subunit WDR4, necessary for the methylation and stabilisation of tRNAs. The largest group (36%) comprises yet uncharacterised proteins; with the remaining potential targets distributed to diverse functional classes ranging from immune response to maintenance and re-modelling of the extracellular matrix (ECM). Interestingly, among mRNAs down-regulated at least by 1.5-fold we found 6 molecules (TBC1D2/Armus, ASAP2, LDLR, M6PR, NKD2 and Rab32) that were shown to play a role in membrane trafficking (**Table 1** **, Table S1**).

### Trafficking-related Potential Targets are Shared by miR-17 Seed Family

Three of the potential miR-17 targets are cargo receptors. LDLR (low density lipoprotein receptor) is necessary for internalisation of LDL maintaining cholesterol homeostasis [44]. M6PR (mannose-6-phosphate receptor) is responsible for the delivery of soluble lysosomal proteins from the trans-Golgi to lysosomes [45]. NKD2 (Naked2: Protein naked cuticle homolog 2) is necessary for targeting TGF-μ to the basolateral membrane of polarised cells [46]. ASAP2 (Arf-GAP with SH3 domain, ANK repeat and PH domain-containing protein 2) and TBC1D2 (TBC1 domain family member 2A)/Armus were shown to act as GAPs for ARF [47] and Rab GTPases [30], respectively. Rab32 is a small GTPase that regulates the formation of autophagic vacuoles [48] and mitochondrial dynamics [49]. Transcripts encoding TBC1D2/Armus, M6PR and LDLR have one, three and five seed-matched sites in their 3′UTRs, respectively. Interestingly, ASAP2 contains one seed-matched site in 3′UTR and three of such sites – in the coding region, but is not computationally predicted to be a target of miR-17. Using bioinformatics, we checked whether trafficking-related potential targets of miR-17 could potentially be regulated by miRNAs bearing the same or similar seed sequence as miR-17. We analysed the members of miR-17-92 cluster as well as two related clusters: miR-106a-363 and miR-106b-25 [14]. TBC1D2 and LDLR turned out to be predicted targets of all miRNAs that share the same seed sequence as miR-17 (**Table 2**). In addition, LDLR is potentially targeted by miR-19a and -19b, which have a different seed sequence than miR-17. M6PR is targeted by the same miRNAs as LDLR; and ASAP2 could be targeted only by miR-19a and -19b. Rab32 and NKD2 did not appear to be targeted by any of the analysed miRNAs (**Table 2**).

**Table 2:** Computational prediction of the trafficking regulators as putative targets of miR-17-92, miR-106a-363 and miR-106b-25 cluster miRNAs.

	TBC1D2	ASAP2	LDLR	M6PR	RAB32	NKD2	miRNA seedsequence
miR-17^a^	+^b^	−	+	+	−	−	5'-AAAGUGC-3'
miR-20a	+	−	+	+	−	−	
miR-20b	+	−	+	+	−	−	
miR-93	+	−	+	+	−	−	
miR-106a	+	−	+	+	−	−	
miR-106b	+	−	+	+	−	−	
miR-18a	−	−	−	−	−	−	5'-AAGGUGC-3'
miR-18b	−	−	−	−	−	−	
miR-92a	−	−	−	−	−	−	5'-AUUGCAC-3'
miR-25	−	−	−	−	−	−	
miR-363	−	−	−	−	−	−	
miR19a	−	+	+	+	−	−	5'-GUGCAAA-3'
miR19b	−	+	+	+	−	−	
miR-320a	−	−	−	−	−	−	5'-AAAGCUG-3'

a Nomenclature of miRNAs as it is used in miRBase database.

b Computational gene prediction by miRanda, Diana-microT and TargetScanHuman algorithms.

### Validation of TBC1D2/Armus as miR-17 Target

**Figure 1:**
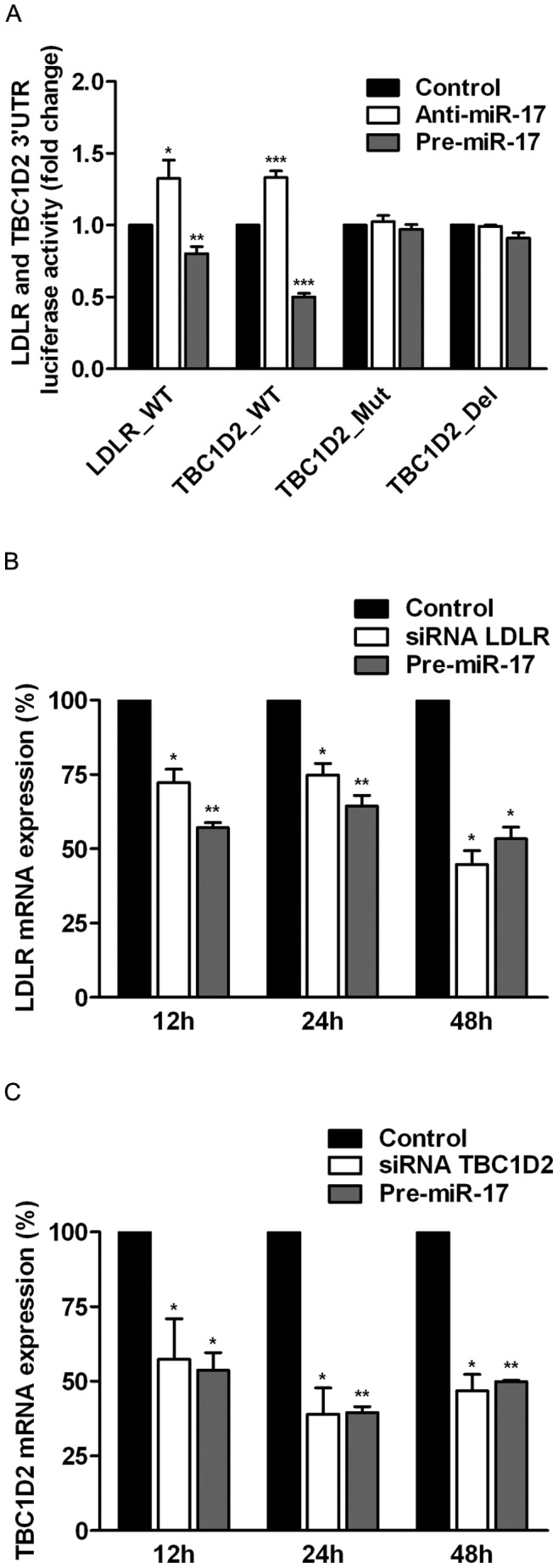
TBC1D2 and LDLR are directly targeted by miR-17. (**A**) miR-17 targets 3′UTRs of TBC1D2 and LDLR as determined by a luciferase reporter assay. HeLa cells were co-transfected with the reporters containing wild-type 3′UTRs of LDLR and TBC1D2 and mutated 3′UTR of TBC1D2, Pre-miR-17 and Anti-miR-17. Luciferase activity was measured 24 h following co-transfection. The activity of luciferase for each experiment was normalized to the activity of the control samples, co-transfected with the respective reporter vector and control Anti-miR or Pre-miR (see **Methods**). The bars show mean fold changes of luciferase activity and the error bars show s.e.m. derived from 3 independent experiments. Over-expression of miR-17 decreased the expression level of LDLR (**B**) and TBC1D2 (**C**) mRNAs as determined by qRT-PCR. The relative expression levels of TBC1D2 and LDLR after treatment with siRNAs and Pre-miR-17 were normalized to the expression level when control siRNAs and control Pre-miR were transfected (see **Methods**). Bars show mean of the relative mRNA expression levels and error bars show s.e.m. derived from 2 independent experiments. *, p≤0,05; **, p≤0,01; ***, p≤0,001.

**Figure 2:**
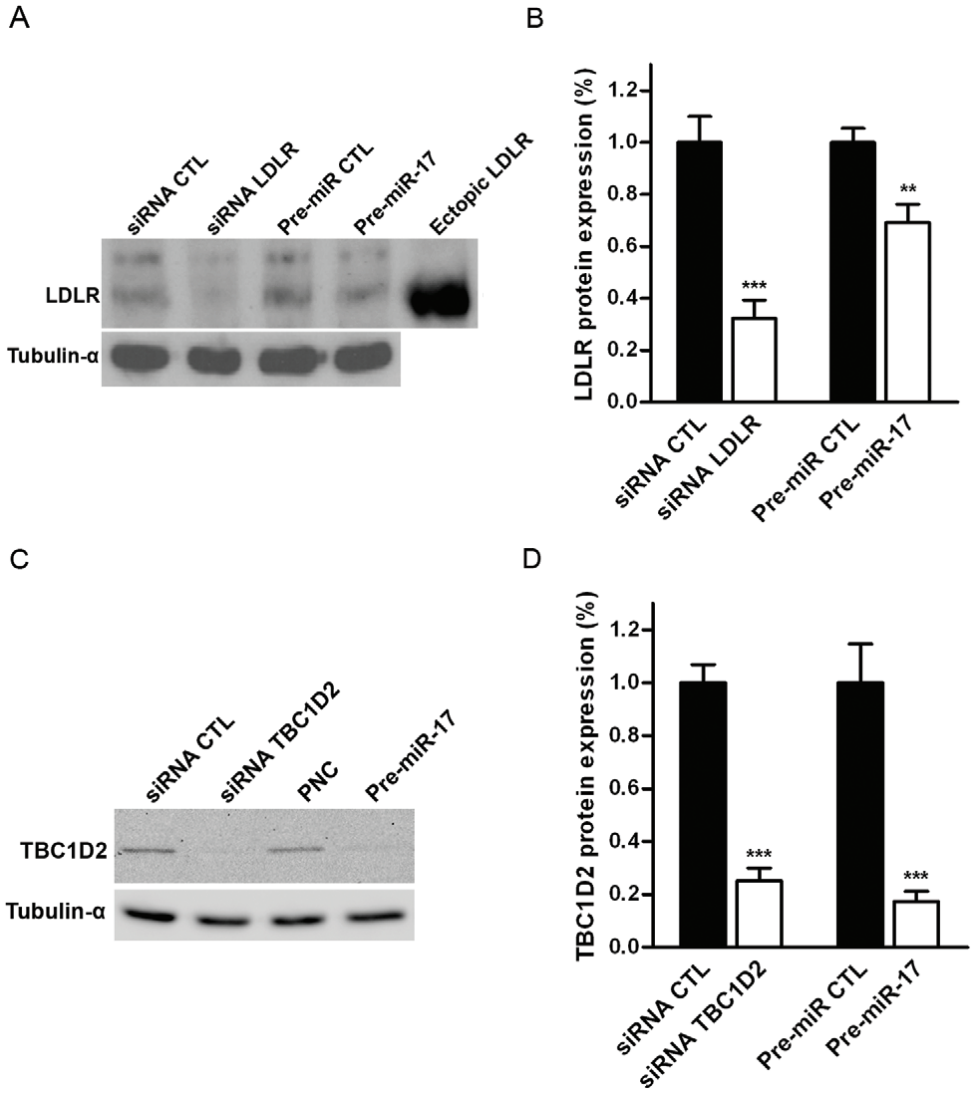
Expression of TBC1D2/Armus is inhibited by miR-17 over-expression. Expression of LDLR (**A, B**) and TBC1D2/Armus (**C, D**) in HeLa cells 48 h after the transfection with siRNAs targeting LDLR and TBC1D2/Armus, and Pre-miR-17. α**-**tubulin was used as a loading control. The sample with ectopically expressed full-length non-tagged LDLR was collected after 24 h of transfection. The expression levels of LDLR (**B**) and TBC1D2/Armus (**D**) after treatment with the respective siRNAs and Pre-miR-17 were normalized to the expression levels of these proteins when cells were treated with the respective negative controls (relative expression). Bars indicate means of relative expression and the error bars indicate s.e.m. derived from at least 3 independent experiments. **, p≤0,01; ***, p≤0,001.

For further validation of trafficking-related targets of miR-17 TBC1D2/Armus and LDLR were selected. We cloned 3'UTRs of these molecules into a dual-luciferase reporter, and measured that the over-expression or inhibition of miR-17 had a strong influence on the luciferase signal from both constructs, indicating that TBC1D2 and LDLR are direct novel targets of miR-17 (**Fig. 1A**). In addition, we mutated or deleted the whole seed site in 3′UTR of TBC1D2 (see **Methods**) to check, whether it is functional in binding to miR-17. Indeed, no changes in luciferase signal were detected with the mutated constructs (**Fig. 1A**). Similar data were obtained when other members of miR-17 seed family, namely, miR-20a and miR-93, were over-expressed. In difference, no changes in luciferase signal were obtained with the over-expressed miR-92a (**Fig. S3**). In contrast to TBC1D2, we did not produce mutants and deletion mutants in 3′UTR of mRNA encoding LDLR due to the presence of five predicted seed matching sites of miR-17. That remains to be done in a follow-up studies in order to understand whether all of these sites are necessary for miR-17 interaction to mRNA of LDLR. Next, qRT-PCR showed that mRNAs levels of LDLR and TBC1D2 were both reduced when miR-17 was over-expressed or when RNAi was performed. The measurements performed after 12 h, 24 h and 48 h of incubation with siRNAs and Pre-miR-17 revealed that the expression of TBC1D2 and LDLR changed in a similar pattern under both conditions (**Fig. 1B, C**). 60% of mRNA encoding TBC1D2 remained after 12 h of incubation and less than 40% - after 48 h of incubation with either siRNA targeting TBC1D2 or Pre-miR-17. Depletion of mRNA encoding LDLR is less efficient after 12 h and 24 h of incubation, but is reduced to nearly 50% after 48 h of incubation with siRNA targeting LDLR or Pre-miR-17 (**Fig. 1B**). Importantly, changes in mRNA levels determined by qRT-PCR and microarray experiments correlate well at all time points tested (**Table 1** **, Table S1**). Next, we checked how the over-expression of miR-17 influence LDLR and TBC1D2/Armus protein expression level by Western Blotting (WB) (**Fig. 2**). As primary antibodies recognising LDLR produce several bands, we have included the sample with ectopically expressed non-tagged LDLR. When compared to mRNA expression level, LDLR protein level changed in a different way when cells were treated with siRNA targeting LDLR or Pre-miR-17. After incubation with siRNA targeting LDLR for 48 h more than 70% of LDLR protein was depleted, whereas about 30% of LDLR reduction was observed in cells treated with Pre-miR-17 (**Fig. 2A, B**). It is not clear why miR-17 over-expression leads to quite efficient depletion of LDLR mRNA, but less efficient depletion of the protein itself. Potentially, down-regulation of multiple other targets might influence LDLR protein translation, stability or degradation. In contrast, TBC1D2/Armus expression was reduced by 70%–80% irrespective of the method used (**Fig. 2C, D**). A strong depletion of TBC1D2/Armus protein following miR-17 over-expression indicates the functional significance of TBC1D2/Armus in regulatory processes guided by miR-17.

### Down-regulation of TBC1D2/Armus Inhibits Endocytic Trafficking

**Figure 3:**
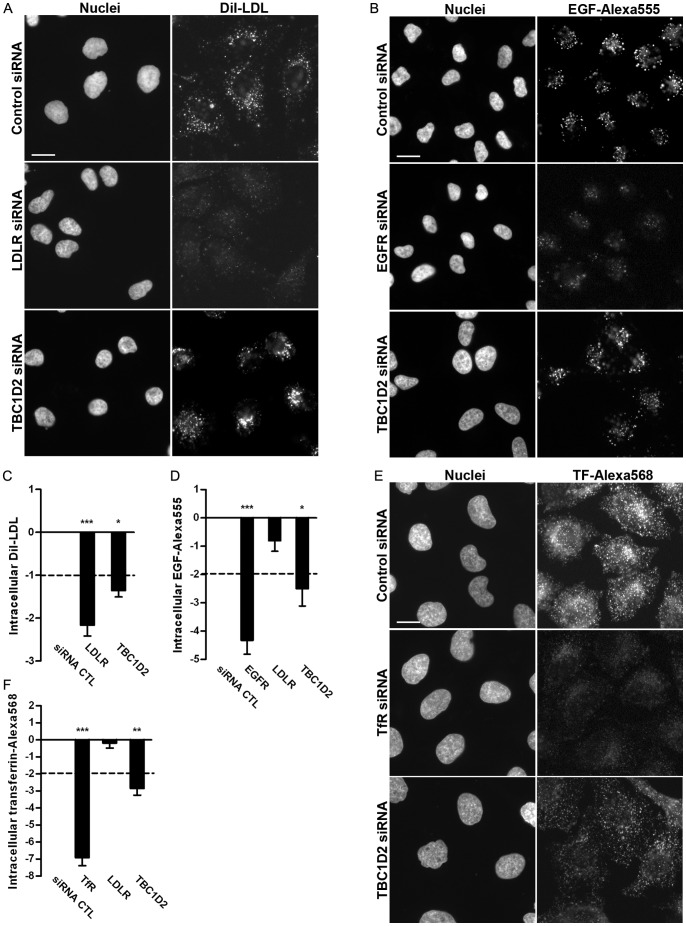
Down-regulation of TBC1D2/Armus inhibits endocytic trafficking. Localisation of intracellular DiI-LDL (**A**), EGF-Alexa555 (**B**) and transferrin-Alexa568 (**E**) in HeLa cells when TBC1D2/Armus (lower rows), the positive controls (middle rows) and the negative control (upper rows) were down-regulated for 48 h. Scale bar represents 20 µm. Fluorescent microscopy-based quantitative assay of LDL (**C**) and EGF (**D**) and transferrin (**F**) trafficking in HeLa cells following the depletion of TBC1D2/Armus for 48 h. Normalized intracellular Dil-LDL, EGF-Alexa555 and transferrin-Alexa568-specific fluorescence intensities are plotted on Y axes. The dashed lines indicate thresholds (see **Methods**) to separate changed from non-changed trafficking for each cargo. Bars show the mean values of three independent experiments and error bars show s.e.m. *, p≤0,05; **, p≤0,01; ***, p≤0,001.

After having validated TBC1D2/Armus as a target of miR-17, we further analysed whether this protein, a GAP for Rab7a, in mammalian cells [30], plays a role in endocytic trafficking of various cargoes. To address this we performed fluorescence microscopy-based LDL, EGF and transferrin internalization assays (see **Methods**). Cells were incubated with the respective ligands, chased at 37°C and intracellular cargoe specific fluorescence was measured on a single cell-basis. After statistical data analysis, the thresholds for individual ligands were set at different levels (see Methods) as they are ligand-specific, due to different fluorescence intensities of the internalised cargoes. As positive controls siRNAs targeting LDLR (see **Fig. 1B** **,** **Fig. 2A, B**), EGFR and TfR were used. siRNAs targeting EGFR and TfR were validated by the producer (see Methods), and, indeed, nearly 50% of depletion of mRNAs encoding EGFR and TfR was obtained after 48 h of incubation as judged by RT-PCR (**Fig. S4**). Down-regulation of LDLR, EGFR and TfR for 48 h strongly reduced the uptake of LDL, EGF and transferrin, respectively (**Fig. 3**). Depletion of TBC1D2/Armus reduced the overall amount of intracellular Dil-LDL specific fluorescence (**Fig. 3A, C**). Intracellular Dil-LDL, although reduced, accumulated in juxtanuclear located as well as in punctuate cytoplasmic structures (**Fig. 3A**). Similar observations have been described for TBC-2, an orthologue of TBC1D2 in *C. elegans* [50]. Internalised GFP tagged yolk protein (vitellogenin), which shares a sequence homology and functional similarity with LDL [51], also accumulates in vesicles concentrating in a juxtanuclear region in tbc-2 mutants [52]. Depletion of TBC1D2/Armus inhibited internalisation of EGF, which, similar to LDL, can be degraded in lysosomes [53], [54] (**Fig. 3B, D**). The ligand preferentially localized to juxtanuclear structures after depletion of TBC1D2/Armus, while in cells treated with the negative control EGF was distributed to cytoplasmic structures. These results supports the previous observation [30] that TBC1D2/Armus acts as a GAP for Rab7a GTPase.

We further analysed whether the down-regulation of TBC1D2/Armus influences internalisation of the re-cycling cargo transferrin [55], [56]. Similar to other cargoes, depletion of TBC1D2/Armus induced a reduction in levels of intracellular transferrin (**Fig. 3E, F**), which supports a potential role of this Rab GAP in the transferrin cycle [57]–[59] and opens a possibility of this GAP being active on other Rabs, for instance Rab5. In contrast to LDL and EGF, that were accumulated in juxtanuclear structures (**Fig. 3A, 3B**), no intracellular accumulation of transferrin was observed 15 min after internalisation (**Fig. 3E**). Instead, reduced, but quite evenly distributed transferrin-specific fluorescence in cytoplasmic punctuate structures was recorded. Different from the previous work demonstrating that high expression levels of both activated Rab5 and TfR induces an accumulation of transferrin in enlarged endosomal structures [60], the observed phenotype in our experiments might be due to much lower endogenous levels of TfR and potentially activated Rab5. The latter resembles transferrin internalisation in A431 cells stably expressing low levels of GFP-tagged Rab5 [61]. Alternatively, transferrin transition to a re-cycling juxtanuclear compartment [62] might be impaired. It is not likely that re-cycling of transferrin to PM [63] is inhibited when TBC1D2/Armus is down-regulated.

### Over-expression of the miR-17 Seed Family Inhibits Endocytic Trafficking

We next performed LDL, EGF and transferrin internalisation assays following the over-expression of four miRNAs, that are predicted to target mRNA of TBC1D2/Armus (miR-17, miR-20a, miR-20b, miR-93) and four miRNAs, that are not predicted to target mRNA of TBC1D2/Armus (miR-18a, miR-19a, miR-92a, miR-320a) (**Table 2**). We demonstrated by qRT-PCR (**Fig. S1A**) and dual-luciferase assay using the reporter plasmids with complementary miRNA binding sites (**Fig. S1B**) that not only miR-17, but also other selected RNA oligonucleotides (Pre-miR-20a, Pre-miR-92a, Pre-miR-93 and Pre-miR-320a) are functional to mimic the over-expression of the respective miRNAs. Moderate over-expression levels (6 to 60 times) were reached under these conditions as assessed by qRT-PCR (**Fig. S1A**). All tested miRNAs are endogenously expressed in HeLa cells (data not shown).

**Figure 4:**
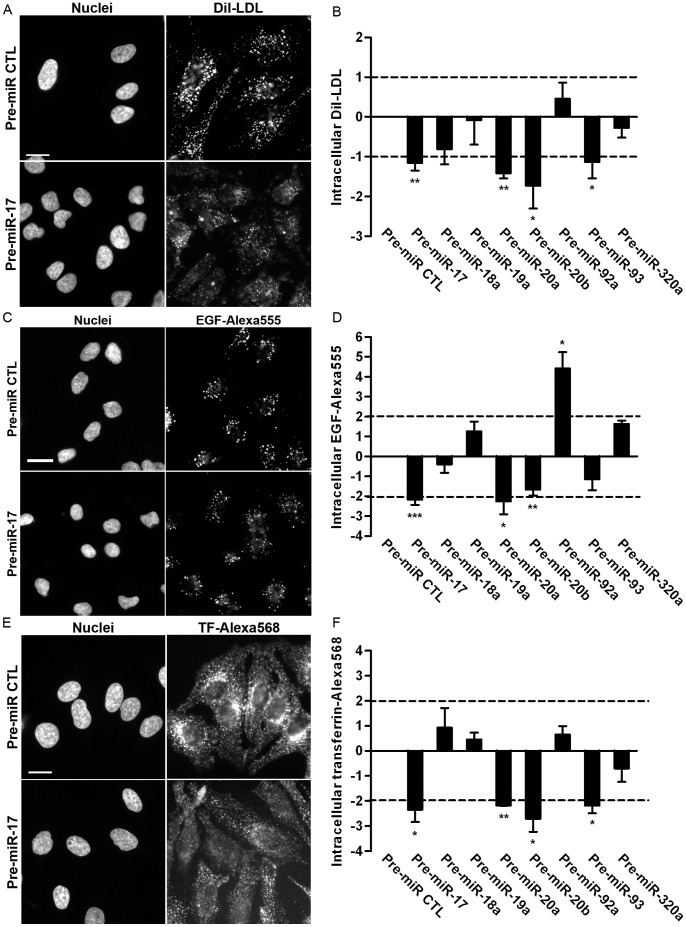
Over-expression of miR-17 seed family inhibits endocytic trafficking. Examples of distribution of intracellular Dil-LDL (**A**), EGF-Alexa555 (**C**) and transferrin-Alexa568 (**E**) following miRNAs over-expression in HeLa cells. Treatment of cells with the negative control miRNA for 48 h had no influence on cargo trafficking, whereas the over-expression of miR-17 family members inhibited trafficking of each tested cargo. Scale bar = 20 µm. Fluorescent microscopy-based quantitative trafficking assays of LDL (**B**), EGF (**D**) and transferrin (**F**) following the 48 h over-expression of miRNAs. Cargo-specific fluorescence intensities were normalized to the negative control and are shown on the Y axes. The positive values show increase and negative values show inhibition of ligand trafficking. The dashed lines indicate respective thresholds (see **Methods**). *, p≤0,05; **, p≤0,01; ***, p≤0,001.

The over-expression of nearly every miRNA, that shares miR-17 seed sequence and potentially targets TBC1D2/Armus (miR-17, miR-20a, miR-20b and miR-93) inhibited trafficking of LDL, EGF and transferrin (**Fig. 4**). Only the over-expression of miR-93 had clearly no effect on EGF internalisation. Normalised intracellular EGF-specific fluorescence stayed slightly below the threshold separating normal and inhibited ligand trafficking (see Methods) when miR-20b was over-expressed (**Fig. 4D**). However, we scored this effect as inhibitory, because of the statistical significance (p-value ≤0,01). Distribution of internalised EGF (juxtanuclear) and transferrin (cytoplasmic structures) under these conditions resembles that of RNAi of TBC1D2/Armus (**Fig. 3B, E**), strengthening the possibility that TBC1D2/Armus might be involved into these processes. In contrast, hardly any accumulation of internalised LDL at the juxtanuclear area was observed (**Fig. 4A**). Presumably, that can be explained by a reduced levels of LDLR – yet another potential target of miR-17 - and, consequently, a reduced amount of receptors at the cell surface available for the ligand. Curiously, LDLR might be also targeted by miR-19a at five predicted positions at 3′UTR, albeit at different sites when compared to miR-17 (data not shown). However, over-expression of miR-19a caused no change in LDL internalisation (**Fig. 4B**) indicating that either the predicted interactions of miR-19a and mRNA of LDLR are non-functional or they have little cellular consequences. The over-expression of miR-18a, miR-19a, miR-92a and miR-320a, the molecules predicted not to target mRNA of TBC1D2/Armus, had no effect on trafficking of LDL, EGF and transferrin (**Fig. 4** **,** **Table 2**) with the exception of miR-92a, which accelerates EGF internalisation when over-expressed (**Fig. 4D**). Normalised intracellular EGF- and LDL-specific fluorescence levels were close to the threshold values (see Methods) when miR-320 and miR-18a are over-expressed, respectively. However, in contrast to miR-20b, the effects turned out to be statistically non-significant (p-value >0,05). Interestingly, miR-320a was recently reported to target and reduce surface expression level of TfR [64]. Potentially, TfR is reduced to such levels that no measurable changes in transferrin trafficking could be recorded in our assay or different conditions need to be tested. To investigate in depth how EGF, LDL and transferrin progression through endocytic pathway is impaired when expression of miR-17 seed family and its target TBC1D2/Armus is altered remains to be analysed in follow-up studies by time-resolved co-localisation experiments and live-cell imaging.

### Over-expression of TBC1D2/Armus GAP Domain Rescues EGF Trafficking Inhibition Induced by miR-17 Over-expression

**Figure 5:**
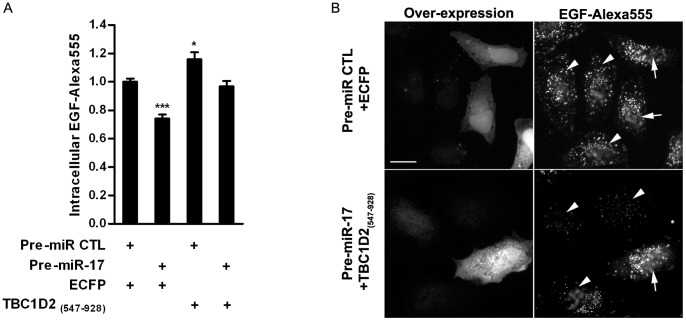
Over-expression of TBC1D2/Armus _547–928_ rescues inhibition of EGF trafficking caused by miR-17 over-expression. Quantification (**A**) and examples (**B**) of over-expressed TBC1D2/Armus_547–928_ counteracting EGF trafficking inhibition. (**A**) Average EGF specific fluorescence is normalised to 1 when Pre-miR and ECFP are over-expressed (Y axis). *, p≤0,05; ***, p≤0,001. Scale bar in (**B**) = 20 µm, arrows indicate transfected cells, arrowheads indicate non-transfected cells.

Next, we have tested whether the down-regulation of TBC1D2/Armus accounts for inhibition of endocytic trafficking caused by the over-expression of miR-17 seed family. Due to similar phenotypes induced under both conditions (**Fig. 3B** and **Fig. 4C**) we have chosen to analyse EGF trafficking. We have tested C-terminally located GAP domain of TBC1D2/Armus_547–928_, previously used for rescue experiments [30]. HeLa cells were initially transfected with Pre-miR-17 and after 24 h of incubation the plasmid encoding a recombinant TBC1D2/Armus_547–928_ was transfected. Following additional 24 h of incubation, intracellular EGF was quantified after 15 min of internalisation (see **Methods**). Over-expression of miRNA negative control and soluble CFP has hardly any influence on EGF trafficking and it was set to 1 (**Fig. 5A, B**). The over-expression of miR-17 induces nearly 30% inhibition of EGF trafficking in the presence of CFP. Over-expression of TBC1D2/Armus_547–928_ leads to little (around 20%) acceleration of EGF trafficking. Combined over-expression of miR-17 and TBC1D2/Armus_547–928_ nearly completely rescues EGF inhibition induced by over-expression of miR-17 (**Fig. 5A**). Our results show that the inhibitory effect on EGF trafficking caused by elevated levels of miR-17 involves TBC1D2/Armus.

### Over-expression of miR-17 Seed Family Reduces Proliferation of HeLa Cells

miR-17 and related miRNAs were shown to change cell proliferation, however, different effects were reported in different cellular contexts [24]. We set a microscopy-based apoptosis and mitosis assay to test the performance of over-expressed miR-17 and related miRNAs in HeLa cells. miRNAs were over-expressed for 24 h to 72 h, cell nuclei were stained and live cell imaging was performed every 24 h. After nuclei segmentation of live cells with an accuracy of nearly 96%, the cells were automatically classified into four different classes: interphase, mitosis, apoptosis, and so called “artefacts” using the extended approach of Harder *et al* [36]. We could classify interphase cells with an accuracy of 97.6% and mitotic cells - with an accuracy of 82% (**Fig. S5A**, see **Methods**). Down-regulation of INCENP (inner centromere protein) with siRNAs for 48 h significantly reduced numbers of mitotic cells in population (**Fig. S5B**) in agreement to the published data [65]. As an additional control, miR-320a was that is known to inhibit proliferation in A549 cells [64]. In agreement to that, numbers of total and mitotic HeLa cells transfected with Pre-miR-320a were reduced (**Fig. S5B**). In contrast to down-regulation of INCENP, no multi- or binucleated cells were detected (these cells were classified as “artefacts” in this work (**Fig. S5A**)) when miR-320a was over-expressed. Over-expression of the selected miRNAs containing miR-17 seed sequence (miR-17, miR-20a and miR-93) for 48 h reduced numbers of mitotic cells, whereas, the over-expression of miR-92a, which bears a different seed sequence, had no effect (**Fig. S5B**). In addition, change of total cell numbers for every tested Pre-miR during the assay was calculated and it was found that the reduced or not increased total cell numbers correspond to reduced numbers of mitotic cells (**Fig. S5C**). For instance, reduction in total cells numbers was observed when miR-17 and miR-93 were over-expressed for 24–72 h, whereas over-expression of miR-92a did not show any influence on cell proliferation.

In addition, RNAi experiments (**Fig. 3**) indicate that, most likely, TBC1D2/Armus is not involved in regulation of HeLa proliferation as no significant changes in cell numbers and cells undergoing mitosis were observed in microscopy-based assays when this proteins was depleted. In addition, TBC1D2/Armus was previously reported not to influence cell cycle progression in HeLa cells [65].

## Discussion

The roles of miR-17-92 are primarily attributed to targeting of diverse regulators of the cell cycle, apoptosis and transcription factors [28] and either increasing or reducing cell proliferation depending on the cellular context [24]. For instance, the over-expression of miR-17 induces proliferation in HEK293T cells [28]. Accordingly, down-regulation of miR-17 and miR-20a inhibits growth of lung cancer cells [66]. In contrast, down-regulation of miR-20a increases proliferation of K562 [67]. In our study, members of the miR-17 seed family reduces numbers of mitotic and total numbers of HeLa cells when over-expressed (**Fig. S5B, C**). Similar observations are reported for breast cancer cells [43] and for non-tumour endothelial cells following miR-17 over-expression [68].

Recent work reveals that the miR-17/20 cluster can regulate cellular microenvironment through modulation of secretion efficiency [69]. In this study we have exploited fluorescence microscopy-based assays to show that miRNAs sharing the seed sequence of miR-17 potentially regulate endocytic trafficking when over-expressed (**Fig. 4**). The observed effect is, most likely, specific as other trafficking-related cellular functions, such as morphology of the Golgi complex, are not changed under these conditions (data not shown). Notably, no effects are seen with over-expressed miR-18, despite only one nucleotide difference from the miR-17 family within the seed sequence. The novel function of miR-17 seed family is attributed to several trafficking regulators, which we have identified by mRNA expression profiling (**Table 1**, **Table S1** and **Fig. S2**). These experiments were performed under conditions of transient miR-17 over-expression (reaching up to 30-fold over-expression, see **Fig. S1A**). The presence of the endogenously expressed miR-17 might possibly account for somewhat lower numbers of the down-regulated mRNAs compared with the experiments where potential targets have been searched in the absence of endogenous expression of the respective miRNAs [6], [70]. Despite that, analyzing the over-expression of miRNAs endogenously present in cells is a valid approach to induce significant down-regulation of the targets and cause the expected cellular effects [71]. A recent proteomics study [72] also identified several trafficking-related proteins (e.g., Rab14) when members of miR-17-92 cluster were down-regulated in SBC-3 lung cancer cells. When miR-20a, belonging to miR-17 seed family is down-regulated, an increase in the expression of ARHGAP12 (Rho GTPases activating protein 12) and TSG101, a member of ESCRT1 complex was observed in this study. TSG101 has been identified as a potential target also in our study, albeit, the change of TSG101 expression level in mRNA profiling experiments is too low to score it as a strong hit (data not shown). That potentially accounts for the differences among applied large-scale techniques, cellular response to modulation of miRNA levels as well as differences in individual miRNA abundance in various cells.

Strong reduction of mRNA expression and the presence of predicted seed matching sites served to narrow-down our search to four trafficking-related proteins (TBC1D2, M6PR, ASAP2 and LDLR). Two of them, LDLR and TBC1D2/Armus, were chosen for validation by luciferase reporter assays (**Fig. 1A**), qRT-PCR (**Fig. 1B, C**), WB (**Fig. 2**) and RNAi (**Fig. 3**). RNAi of TBC1D2/Armus has broad effects on endocytic trafficking and the protein expression is effectively reduced by miR-17 over-expression (**Fig. 2C, D**). Armus is thus a bona fide target of miR-17, which expression changes have broad cellular consequences. To check that, we performed a rescue experiment with a GAP domain of TBC1D2/Armus over-expressed. Indeed, nearly a complete rescue of inhibition of EGF internalisation caused by miR-17 over-expression is obtained by over-expression of Armus GAP domain (**Fig. 5**), indicating an important role of this protein in miR-17 activity.

TBC1D2/Armus has been recently shown to be a GAP for Rab7 in mammalian cells [30]. However, the same study showed that the over-expression of TBC1D2/Armus RabGAP induced redistribution of Rab5-positive endosomes in keratinocytes. We show here that the depletion of TBC1D2/Armus in Hela cells by 70–80% (**Fig. 2C, D**) induces trafficking inhibition not only of cargo degraded in lysosomes, but also that of the re-cycling cargo transferrin (**Fig. 3E, F**). As it is unlikely that Rab7 is involved in the transferrin cycle [57], [59], [73], TBC1D2/Armus might have broader activity as a GAP only for Rab7, but further work is necessary to test whether Rab5 or also other Rabs are regulated (directly or indirectly) by TBC1D2/Armus. Reduced amounts of intracellular EGF, LDL and transferrin are observed at a given time point following the depletion of TBC1D2/Armus. That could mean perturbation in uptake, internalisation or increased re-cycling, but these possibilitiesalso remain to be resolved. The orthologue of TBC1D2/Armus in *C. elegans* (TBC-2) acts as a GAP for both Rab7 and Rab5, regulating storage of yolk protein in larval survival and cell corpse clearance [50], [52], [74]. It is possible that mammalian and *C.elegans* proteins preserve their GAP substrate specificity, but a direct comparison is difficult due to a modest homology level (around 29%) between TBC1D2/Armus and TBC-2.

Our data supports the previous observations that this RabGAP regulates internalisation of E-cadherins [30]. Upon disassembly of cell-to-cell contacts, E-cadherin is targeted to lysosomes for degradation in a Rab7-specific manner [75]. That frequently occurs during epithelial to mesenchymal transitions in development and metastasis [76], [77]. On the other hand, availability of E-cadherin on basolateral cell membrane is also regulated by re-cycling of internalised pool [78]. In any case, internalised E-cadherin may enter early endosomal compartment in a Rab5-dependent manner [79], and TBC1D2/Armus might exert the control at this level if acting as a GAP for Rab5. On the other hand, some targets of miR-17 family, namely STAT3 (Signal transducer and activator of transcription 3) and MAPK14 (Mitogen-activated protein kinase 14), regulate expression of E-cadherin directly [29]. miR-17 family might contribute to precisely regulate complex E-cadherin surface expression in a traffic-dependent and independent manner; but the individual contribution of diverse pathways remains to be analysed.

Over-expression of miR-17 effectively reduces expression of LDLR mRNA (**Fig. 1B**), but expression of the protein itself is reduced only by 30% under these conditions (**Fig. 2B**), and might have little relevance in a physiological context. On the other hand, 3′UTR of LDLR mRNA contains multiple seed matching sites of miR-17, which usually indicates targeting by a given miRNA. In addition, the over-expression of miR-17 induces little appearance of intracellular LDL (**Fig. 4A**). The latter resembles the phenotype of LDLR RNAi (**Fig. 3A**) and indicates that targeting of LDLR by miR-17 is physiologically significant. Regulation of LDLR expression level by miR-17 may participate in regulation of transport of lipid and lipidofilic compounds into cell, energy production, homeostasis of vitamins and hormones, and membrane synthesis [80]. Furthermore, regulation of LDLR expression at a post-transcriptional level might contribute to molecular requirements specific for internalisation of LDL as large cargo. Indeed, Mettlen and colleagues [81] have reported that the expression level of LDLR directly correlates with the size of clathrin coated pits (CCP), recruitment of adaptors Dab2 and ARH to the PM, and lifetime of productive CCP [82].

Phenotype rescue with the over-expressed GAP domain of TBC1D2/Armus (**Fig. 5**), indicates the importance of this protein in miR-17 mediated regulation, particularly knowing that miR-17 targets numerous other molecules. The functional contribution of other trafficking-related and unrelated targets on miR-17 as a broad regulator of endocytic trafficking remains an exciting area of investigation. Along these lines, the role of miR-17 specifically in uptake, internalisation, degradation and/or re-cycling events needs to be addressed in follow-up studies. On the other hand, by analysing the contribution of trafficking-related targets of miR-17 to other cellular functions regulated by this and related miRNAs will provide valuable insight in understanding a cross-talk among different cellular activities. From the most general point of view, the role of miR-17 in controlling endocytic trafficking matches well to the functions of this miRNA in tumour biology. Elevated levels of miR-17 correlates with reduced endocytosis in cancer cells, resulting in increased levels of, for instance, EGFR on the PM and induced uncontrolled receptor signalling [83] leading to perturbation of proliferation, survival and many other cellular functions.

## Supporting Information

Figure S1 **Tests of** **Pre-miRs functionality.** (**A**) Test of miRNAs over-expression by qRT-PCR. Cells were transfected with the corresponding Pre-miRs and negative controls and qRT-PCR was performed 48 h after the transfection with TaqMan MicroRNA assays. Y axis represents relative changes in the miRNA expression levels, expressed in a logarithmic scale. (**B**) Test of miRNAs over-expression by dual luciferase assay. The assay was performed 48 h after co-transfection of the reporter constructs with the complementary miRNA binding sites and the respective RNA oligonucleotides. Changes in the reporter expression level were quantified as *Renilla*/firefly luciferase ratio normalized against the control sample. In both graphs bars indicate the average values derived from 2–3 independent transfection experiments (for more see Materials and Methods), error bars indicate standard errors. *, p≤0,05; **, p≤0,01; ***, p≤0,001 when compared to mock-transfected cells in (**A**) or to cells transfected only with luciferase reporter plasmid in (**B**).
(TIF)

Figure S2 **Functional annotation of mRNAs, which are down-regulated when miR-17 is overexpressed.**
(TIF)

Figure S3 **TBC1D2 and LDLR are directly targeted by miR-17 seed family.** HeLa cells were co-transfected with the reporters containing wild-type 3′UTRs of LDLR and TBC1D2 and mutated 3′UTR of TBC1D2, Pre-miR-20a, Pre-miR-93 and Pre-miR-92a. Luciferase activity was measured 24 h following the co-transfection. The activity of luciferase for each experiment was normalized to the activity of the control samples, co-transfected with the respective reporter vector and control Pre-miR (see **Methods**). The bars show mean fold changes of luciferase activity and the error bars show s.e.m. derived from 3 independent experiments. **, p≤0,01; ***, p≤0,001.
(TIF)

Figure S4 **Efficiency test of siRNAs targeting EGFR and TfR.** Cells were transfected with the respective siRNAs and the negative control and RT-PCR was performed 48 h after the incubation. Expression of mRNA encoding GAPDH was used for the normalization. Graphs bars indicate the average values derived from 3 independent transfection experiments and error bars indicate standard errors. *, p≤0,05;
(TIF)

Figure S5 **Microscopy-based assay to quantify miRNA influence on cell proliferation.** (**A**) Example images to demonstrate four classes of nuclei considered in the automated image analysis are represented in the upper row. Confusion matrix for classification of these phenotypes using a weighted SVM classifier with fourfold cross-validation and accuracy of the results are demonstrated in the lower row. (**B**) Over-expression of miR-17 seed sequence family reduces the number of mitotic cells. Cells were transfected with the respective Pre-miRs and the negative control, and the fluorescence microscopy based assay to identify mitotic cells was performed in living cells (see **Methods**). Bars show mean values of two independent experiments and error bars show the standard errors of the means. (**C**) Over-expression of the members of miR-17 seed family inhibits cell proliferation. Cells were transfected with Pre-miRs or INCENP siRNA and the total number of cells was quantified after 72 h of continues incubation. Cell numbers of the population transfected with the negative control was set to 1. *, p≤0,05; **, p≤0,01; ***, p≤0,001, (when compare to mock-transfected cells in (**C**)).
(TIF)

Table S1 **mRNA expression analysis under conditions of miR-17-5p over-expression.**
(DOC)
