# Supplementary figures and images for: miR-17-5p Regulates Endocytic Trafficking through Targeting TBC1D2/Armus
Source: PLoS One. 2012 Dec 20;7(12):e52555. doi: 10.1371/journal.pone.0052555 (PMC3527550; doi:10.1371/journal.pone.0052555)

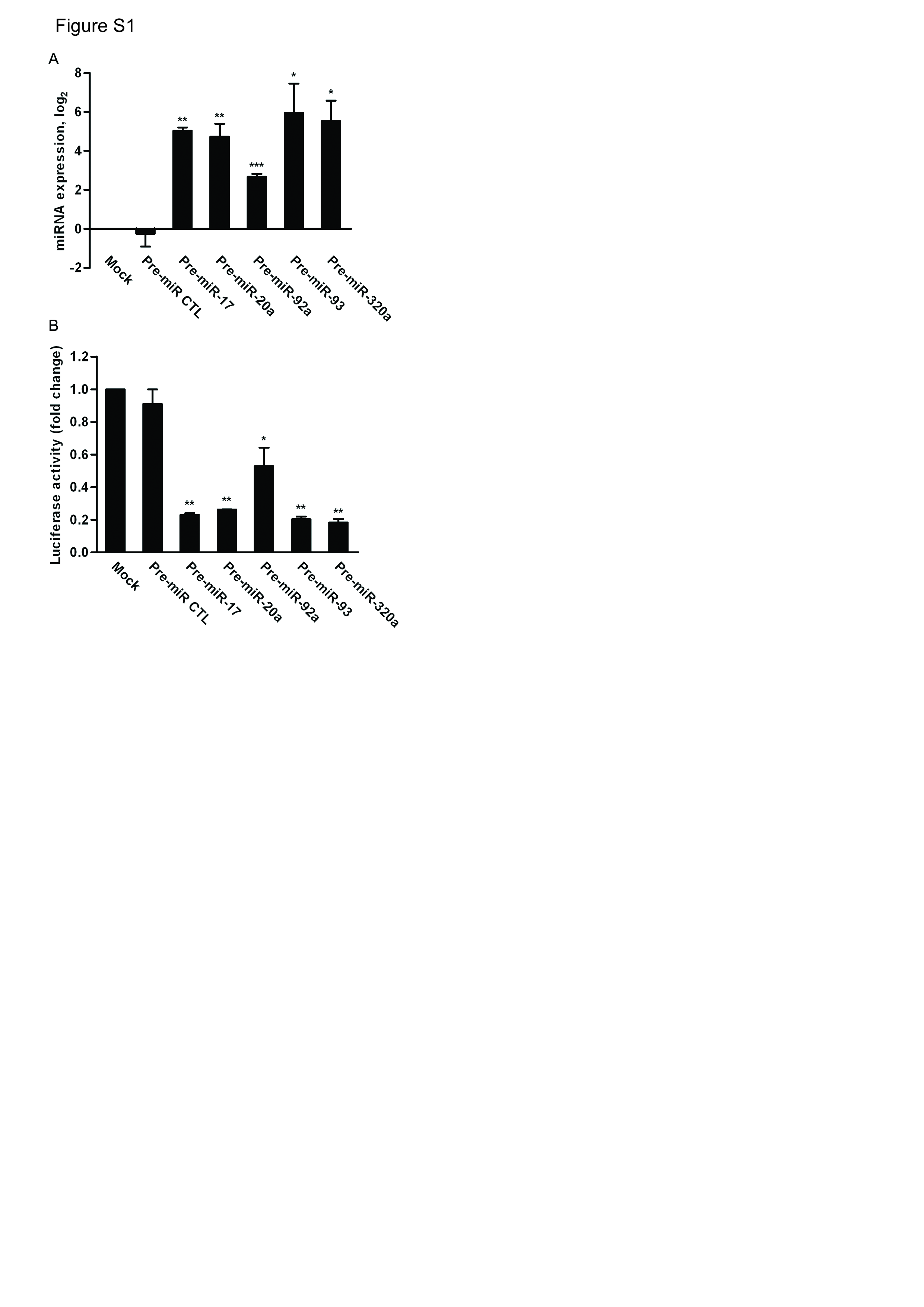

Supplement: Figure S1 — Tests of Pre-miRs functionality. (A) Test of miRNAs over-expression by qRT-PCR. Cells were transfected with the corresponding Pre-miRs and negative controls and qRT-PCR was performed 48 h after the transfection with TaqMan MicroRNA assays. Y axis represents relative changes in the miRNA expression levels, expressed in a logarithmic scale. (B) Test of miRNAs over-expression by dual luciferase assay. The assay was performed 48 h after co-transfection of the reporter constructs with the complementary miRNA binding sites and the respective RNA oligonucleotides. Changes in the reporter expression level were quantified as Renilla/firefly luciferase ratio normalized against the control sample. In both graphs bars indicate the average values derived from 2–3 independent transfection experiments (for more see Materials and Methods), error bars indicate standard errors. *, p≤0,05; **, p≤0,01; ***, p≤0,001 when compared to mock-transfected cells in (A) or to cells transfected only with luciferase reporter plasmid in (B). (TIF) [file pone.0052555.s001.tif]

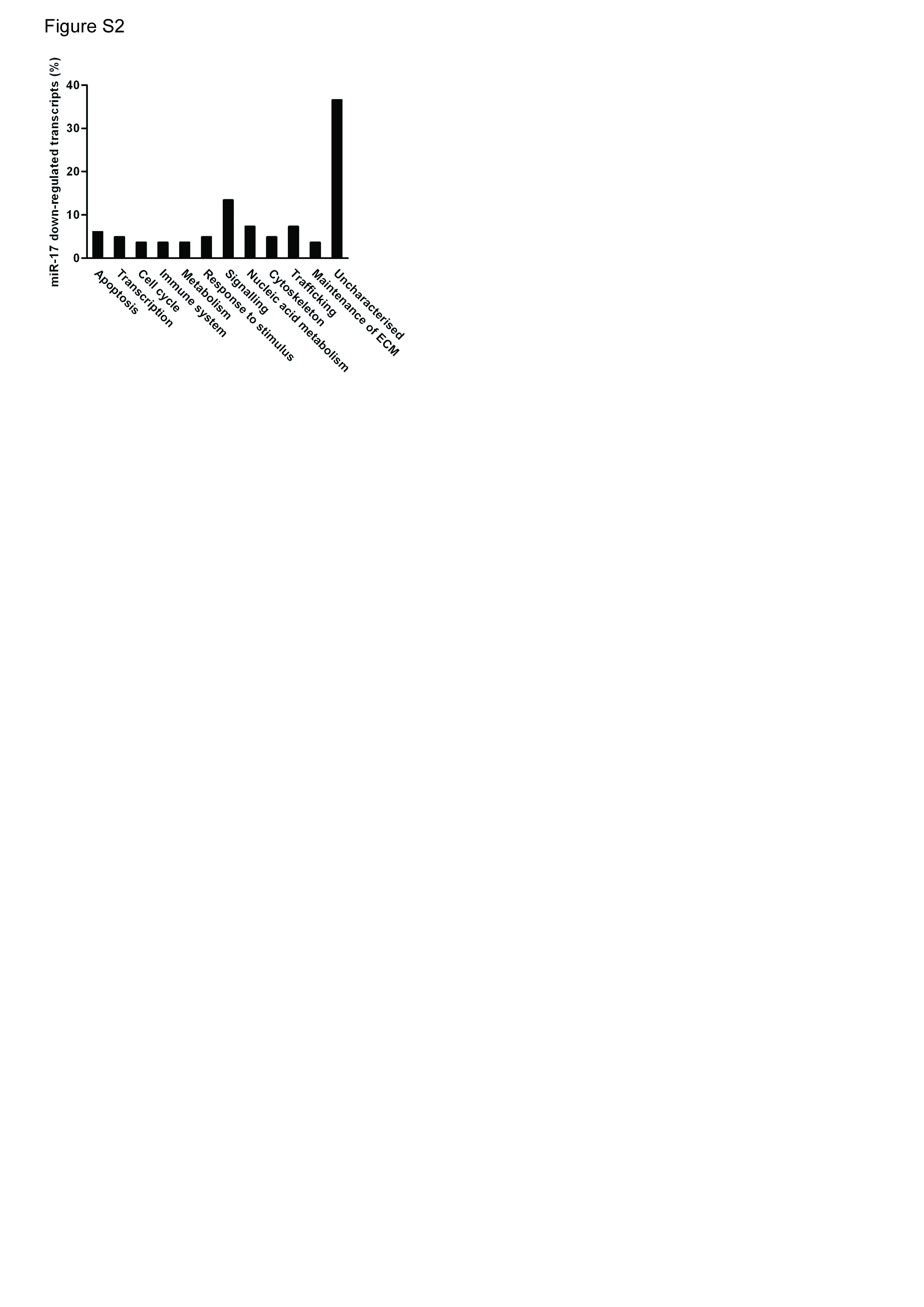

Supplement: Figure S2 — Functional annotation of mRNAs, which are down-regulated when miR-17 is overexpressed. (TIF) [file pone.0052555.s002.tif]

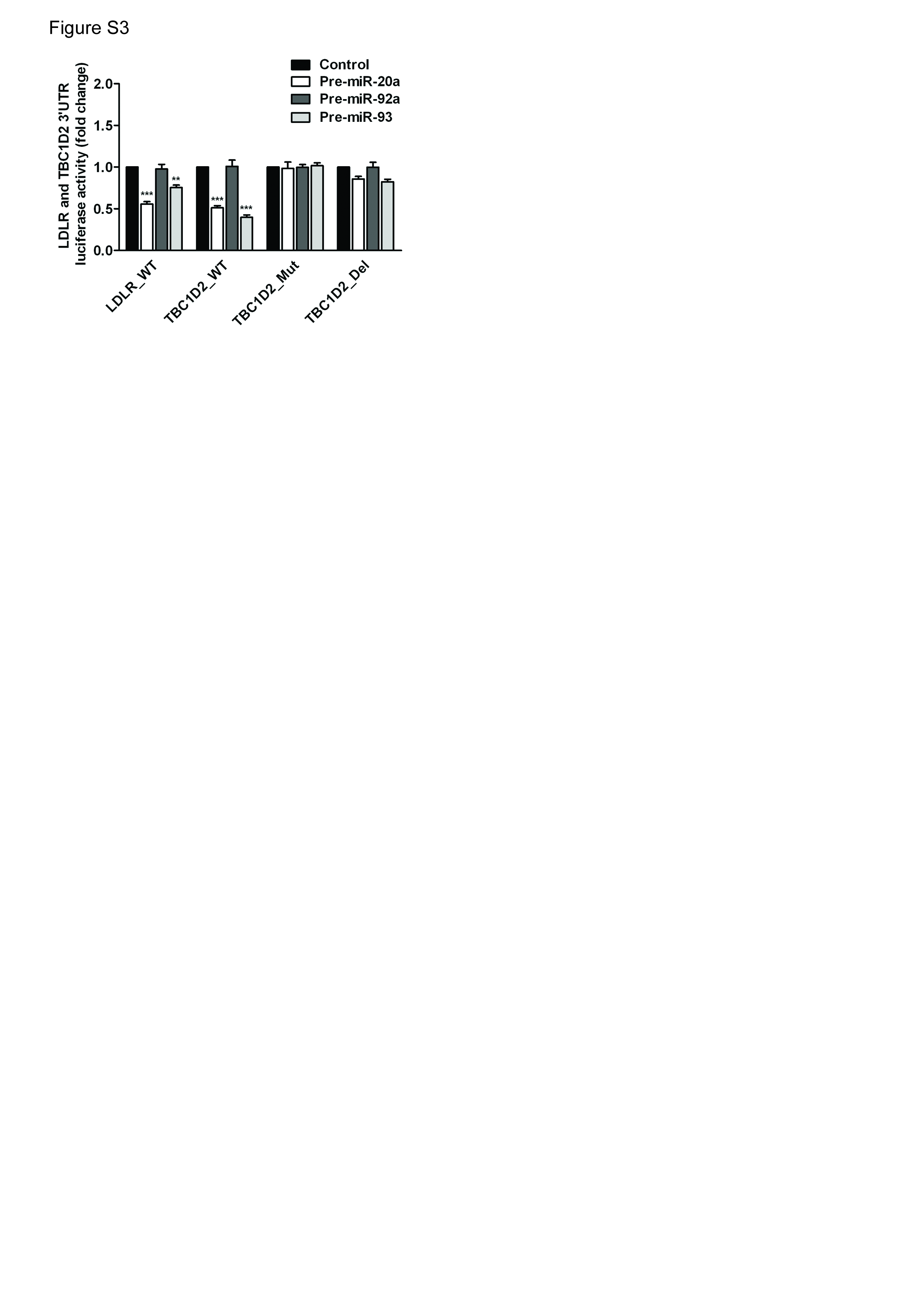

Supplement: Figure S3 — TBC1D2 and LDLR are directly targeted by miR-17 seed family. HeLa cells were co-transfected with the reporters containing wild-type 3′UTRs of LDLR and TBC1D2 and mutated 3′UTR of TBC1D2, Pre-miR-20a, Pre-miR-93 and Pre-miR-92a. Luciferase activity was measured 24 h following the co-transfection. The activity of luciferase for each experiment was normalized to the activity of the control samples, co-transfected with the respective reporter vector and control Pre-miR (see Methods). The bars show mean fold changes of luciferase activity and the error bars show s.e.m. derived from 3 independent experiments. **, p≤0,01; ***, p≤0,001. (TIF) [file pone.0052555.s003.tif]

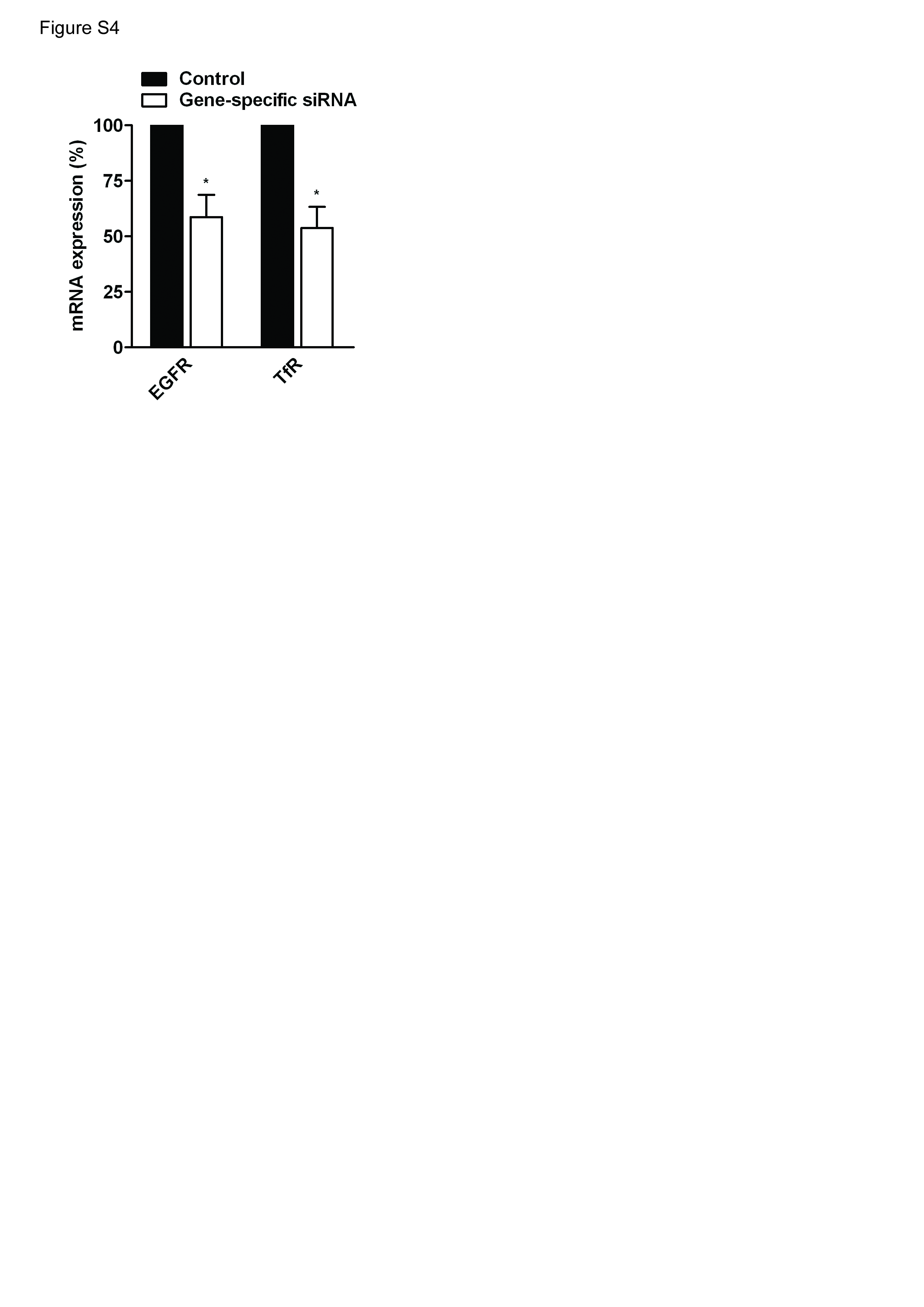

Supplement: Figure S4 — Efficiency test of siRNAs targeting EGFR and TfR. Cells were transfected with the respective siRNAs and the negative control and RT-PCR was performed 48 h after the incubation. Expression of mRNA encoding GAPDH was used for the normalization. Graphs bars indicate the average values derived from 3 independent transfection experiments and error bars indicate standard errors. *, p≤0,05; (TIF) [file pone.0052555.s004.tif]

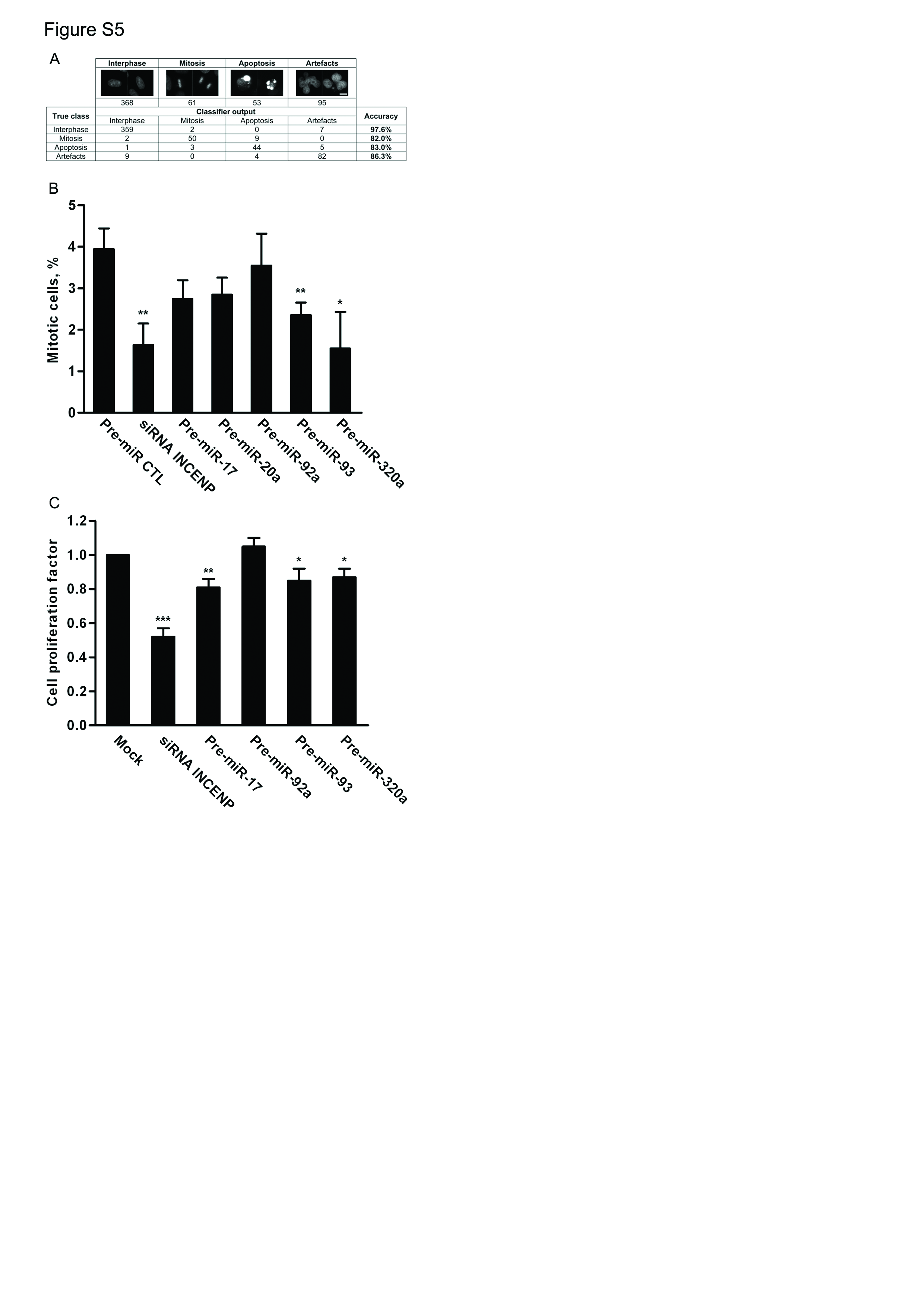

Supplement: Figure S5 — Microscopy-based assay to quantify miRNA influence on cell proliferation. (A) Example images to demonstrate four classes of nuclei considered in the automated image analysis are represented in the upper row. Confusion matrix for classification of these phenotypes using a weighted SVM classifier with fourfold cross-validation and accuracy of the results are demonstrated in the lower row. (B) Over-expression of miR-17 seed sequence family reduces the number of mitotic cells. Cells were transfected with the respective Pre-miRs and the negative control, and the fluorescence microscopy based assay to identify mitotic cells was performed in living cells (see Methods). Bars show mean values of two independent experiments and error bars show the standard errors of the means. (C) Over-expression of the members of miR-17 seed family inhibits cell proliferation. Cells were transfected with Pre-miRs or INCENP siRNA and the total number of cells was quantified after 72 h of continues incubation. Cell numbers of the population transfected with the negative control was set to 1. *, p≤0,05; **, p≤0,01; ***, p≤0,001, (when compare to mock-transfected cells in (C)). (TIF) [file pone.0052555.s005.tif]
