# Supplementary material for: miR-17-5p Regulates Endocytic Trafficking through Targeting TBC1D2/Armus
Source: PLoS One. 2012 Dec 20;7(12):e52555. doi: 10.1371/journal.pone.0052555 (PMC3527550; doi:10.1371/journal.pone.0052555)
Supplement: Table S1 — mRNA expression analysis under conditions of miR-17-5p over-expression. (DOC) [file pone.0052555.s006.doc]

(**A**) 12h after transfection with Pre-miR-17-5p

| 1.5 fold-change | | | | | Number of seed regions | | |
| --- | --- | --- | --- | --- | --- | --- | --- |
| Number | Gene symbol^a^ | Gene accession number^b^ | Gene name | Fold-change^c^ | 5'UTR | CDS | 3'UTR |
| 1 | TGFBR2 | NM_001024847 | Transforming growth factor, beta receptor II (70/80kDa) | -2,0 |  |  | 3 |
| 2 | DAZAP2 | NM_014764 | DAZ associated protein 2 | -1,9 |  |  | 1 |
| 3 | MICA | NM_000247 | MHC class I polypeptide-related sequence A | -1,8 | 1 |  | 1 |
| 4 | TBC1D2 | NM_018421 | TBC1 domain family, member 2 (TBC1D2) | -1,8 |  |  | 1 |
| 5 | HDHD1A | NM_012080 | Haloacid dehalogenase-like hydrolase domain containing 1A | -1,7 |  |  | 3 |
| 6 | NKIRAS1 | NM_020345 | NFKB inhibitor interacting Ras-like 1 | -1,7 |  | 1 | 1 |
| 7 | NETO2 | NM_018092 | Neuropilin (NRP) and tolloid (TLL)-like | -1,7 |  | 2 | 3 |
| 8 | TPRG1LA | NM_182752 | Family with sequence similarity 79, member A | -1,7 |  | 1 | 2 |
| 9 | IL6 | NM_000600 | Interleukin 6 (interferon, beta 2) | -1,7 |  |  |  |
| 10 | FLJ31438 | NM_152385 | Hypothetical protein FLJ31438 | -1,7 |  |  | 3 |
| 11 | ASAP2 | NM_003887 | Development and differentiation enhancing factor 2 | -1,6 |  | 3 | 1 |
| 12 | JAK1 | NM_002227 | Janus kinase 1 (a protein tyrosine kinase) | -1,6 |  | 2 | 2 |
| 13 | TNFRSF21 | NM_014452 | Tumor necrosis factor receptor superfamily, member 21 | -1,6 |  | 1 | 2 |
| 14 | CYBRD1 | NM_024843 | Cytochrome b reductase 1 (CYBRD1) | -1,6 |  |  | 4 |
| 15 | RNH1 | NM_203385 | Ribonuclease/angiogenin inhibitor 1 | -1,6 |  |  | 2 |
| 16 | TMEM9B | NM_020644 | TMEM9 domain family, member B | -1,6 |  |  | 1 |
| 17 | TMEM64 | NM_001008495 | Transmembrane protein 64 | -1,6 |  |  | 3 |
| 18 | FASTK | NM_033015 | Fas-activated serine/threonine kinase | -1,6 |  | 1 | 1 |
| 19 | MKRN1 | NM_013446 | Makorin 1 | -1,6 |  |  | 3 |
| 20 | FYCO1 | NM_024513 | FYVE and coiled-coil domain containing 1 | -1,6 |  |  | 5 |
| 21 | KATNAL1 | NM_032116 | Katanin p60 subunit A-like 1 | -1,6 |  | 2 | 7 |
| 22 | PNKD | NM_022572 | Paroxysmal nonkinesiogenic dyskinesia | -1,5 |  |  | 2 |
| 23 | C21orf25 | NM_199050 | Chromosome 21 open reading frame 25 | -1,5 |  |  | 6 |
| 24 | KIF23 | NM_004856 | Kinesin family member 23 | -1,5 |  |  | 2 |
| 25 | LIMA1 | NM_016357 | LIM domain and actin binding 1 | -1,5 |  | 1 | 2 |
| 26 | FBXO18 | NM_032807 | F-box protein, helicase18 | -1,5 |  |  | 1 |
| 27 | IRAK2 | NM_001570 | Interleukin-1 receptor-associated kinase 2 | -1,5 |  |  | 1 |
| 28 | C2orf29 | NM_017546 | Chromosome 2 open reading frame 29 | -1,5 |  |  |  |
| 29 | OXR1 | NM_181354 | Oxidation resistance 1 | -1,5 |  | 2 | 1 |
| 30 | WDR74 | NM_018093 | WD repeat domain 74 | 1,5 |  |  | 1 |
| 31 | CHPF | NM_024536 | Chondroitin polymerizing factor | 1,5 |  |  |  |
| 32 | TMEM41A | NM_080652 | Transmembrane protein 41A | 1,5 |  |  | 1 |
| 33 | ETF1 | NM_004730 | Eukaryotic translation termination factor 1 | 1,5 |  | 1 | 4 |
| 34 | UBE2O | NM_022066 | Ubiquitin-conjugating enzyme E2O | 1,5 |  | 2 |  |
| 35 | TSC22D3 | NM_004089 | TSC22 domain family, member 3 | 1,5 |  |  | 1 |
| 36 | PPP3R1 | NM_000945 | Protein phosphatase 3, regulatory subunit B, alpha isoform | 1,6 |  | 1 | 2 |
| 37 | LOC285636 | NM_175921 | Hypothetical protein LOC285636 | 1,6 |  | 1 | 2 |
| 38 | ICMT | NM_012405 | Isoprenylcysteine carboxyl methyltransferase | 1,6 |  | 2 | 1 |
| 39 | PMM2 | NM_000303 | Phosphomannomutase 2 | 1,6 |  |  | 1 |
| 40 | BAT2D1 | NM_015172 | BAT2 domain containing 1 | 1,6 |  | 1 |  |
| 41 | PPIF | NM_005729 | Peptidylprolyl isomerase F (cyclophilin F) | 1,7 |  |  |  |

(**B**) 24h after transfection with Pre-miR-17-5p

| 1.5 fold-change | | | | | Number of seed regions | | |
| --- | --- | --- | --- | --- | --- | --- | --- |
| Number | Gene symbol^a^ | Gene accession number^b^ | Gene name | Fold-change^c^ | 5'UTR | CDS | 3'UTR |
| 1 | IL6 | NM_000600 | Interleukin 6 (interferon, beta 2) | -2,1 |  |  |  |
| 2 | JAK1 | NM_002227 | Janus kinase 1 (a protein tyrosine kinase) | -2,0 |  | 2 | 2 |
| 3 | RNH1 | NM_203385 | Ribonuclease/angiogenin inhibitor 1 | -1,9 |  |  | 2 |
| 4 | HDHD1A | NM_012080 | Haloacid dehalogenase-like hydrolase domain containing 1A | -1,9 |  |  | 3 |
| 5 | C9orf152 | NM_001012993 | Chromosome 9 open reading frame 152 | -1,9 |  |  |  |
| 6 | DAZAP2 | NM_014764 | DAZ associated protein 2 | -1,9 |  |  | 1 |
| 7 | TGFBR2 | NM_001024847 | Transforming growth factor, beta receptor II (70/80kDa) | -1,9 |  |  | 3 |
| 8 | TBC1D2 | NM_018421 | TBC1 domain family, member 2 | -1,9 |  |  | 1 |
| 9 | MT2A | NM_005953 | Metallothionein 2A | -1,8 |  |  |  |
| 10 | FAM18B | NM_016078 | Family with sequence similarity 18, member B | -1,7 |  |  | 1 |
| 11 | MICA | NM_000247 | MHC class I polypeptide-related sequence A | -1,7 | 1 |  | 1 |
| 12 | DNAJB6 | NM_005494 | DnaJ (Hsp40) homolog, subfamily B, member 6 | -1,7 |  |  |  |
| 13 | EGR1 | NM_001964 | Early growth response 1 | -1,7 |  |  |  |
| 14 | TNFAIP1 | NM_021137 | Tumor necrosis factor, alpha-induced protein 1 | -1,7 |  | 1 | 2 |
| 15 | FYCO1 | NM_024513 | FYVE and coiled-coil domain containing 1 | -1,7 |  |  | 5 |
| 16 | LOC205251 | NM_174925 | Hypothetical protein LOC205251 | -1,7 |  |  |  |
| 17 | C21orf25 | NM_199050 | Chromosome 21 open reading frame 25 | -1,6 |  |  | 6 |
| 18 | ASAP2 | NM_003887 | Development and differentiation enhancing factor 2 | -1,6 |  | 3 | 1 |
| 19 | ETS2 | NM_005239 | V-ets erythroblastosis virus E26 oncogene homolog 2 | -1,6 |  |  |  |
| 20 | NKIRAS1 | NM_020345 | NFKB inhibitor interacting Ras-like 1 | -1,6 |  | 1 | 1 |
| 21 | FAM46C | NM_017709 | Family with sequence similarity 46, member C | -1,6 |  |  | 4 |
| 22 | TPRG1LA | NM_182752 | Tumor protein p63 regulated 1-like | -1,6 |  | 1 | 2 |
| 23 | WDR4 | NM_018669 | WD repeat domain 4 | -1,6 |  |  | 1 |
| 24 | CYR61 | NM_001554 | Cysteine-rich, angiogenic inducer 61 | -1,6 |  |  |  |
| 25 | DNAJC12 | NM_021800 | DnaJ (Hsp40) homolog, subfamily C, member 12 | -1,6 |  |  |  |
| 26 | MRPL24 | NM_024540 | Mitochondrial ribosomal protein L24 | -1,6 |  |  |  |
| 27 | IRAK2 | NM_001570 | Interleukin-1 receptor-associated kinase 2 | -1,6 |  |  | 1 |
| 28 | BIRC3 | NM_001165 | Baculoviral IAP repeat-containing 3 | -1,6 |  | 2 |  |
| 29 | OBFC2A | NM_022837 | Oligonucleotide/oligosaccharide-binding fold containing 2A | -1,6 |  |  |  |
| 30 | M6PR | NM_002355 | Mannose-6-phosphate receptor (cation dependent) | -1,6 |  |  | 3 |
| 31 | TMEM9B | NM_020644 | TMEM9 domain family, member B | -1,6 |  |  | 1 |
| 32 | PNKD | NM_022572 | Paroxysmal nonkinesiogenic dyskinesia | -1,5 |  |  | 2 |
| 33 | WBSCR22 | NM_017528 | Williams Beuren syndrome chromosome region 22 | -1,5 |  |  | 1 |
| 34 | C9orf40 | NM_017998 | Chromosome 9 open reading frame 40 | -1,5 |  |  | 1 |
| 35 | PHLDA2 | NM_003311 | Pleckstrin homology-like domain, family A, member 2 | -1,5 |  |  |  |
| 36 | RELB | NM_006509 | V-rel reticuloendotheliosis viral oncogene homolog B | -1,5 |  |  |  |
| 37 | MT1A | NM_005946 | Metallothionein 1A | -1,5 | 1 |  |  |
| 38 | MUC1 | NM_001018021 | Mucin 1 | -1,5 |  |  |  |
| 39 | C1QTNF1 | NM_198594 | C1q and tumor necrosis factor related protein ! | -1,5 |  |  |  |
| 40 | PFKP | NM_002627 | Phosphofructokinase, platelet | -1,5 |  |  | 1 |
| 41 | RAB32 | NM_006834 | RAB32, member RAS oncogene family | -1,5 |  |  |  |
| 42 | DCPS | NM_014026 | Decapping enzyme, scavenger | -1,5 |  |  |  |
| 43 | CASP1 | NM_033294 | Caspase 1 | -1,5 |  |  |  |
| 44 | TRIM2 | NM_015271 | Tripartite motif-containing 2 | 1,5 |  | 1 | 1 |
| 45 | BAT2D1 | NM_015172 | BAT2 domain containing 1 | 1,5 |  | 1 |  |
| 46 | TSC22D3 | NM_004089 | TSC22 domain family, member 3 | 1,5 |  |  | 1 |
| 47 | EML4 | NM_019063 | Echinoderm microtubule associated protein like 4 | 1,5 |  | 2 |  |
| 48 | PSAT1 | NM_021154 | Phosphoserine aminotransferase 1 | 1,5 |  |  | 1 |
| 49 | ARPC4 | NM_001024959 | Actin related protein 2/3 complex, subunit 4 | 1,5 |  | 1 | 1 |
| 50 | ARID5B | NM_032199 | AT rich interactive domain 5B (MRF1-like) | 1,5 |  |  |  |
| 51 | SLC11A2 | NM_000617 | Solute carrier family 11, member 2 | 1,6 |  |  | 2 |
| 52 | PLCXD1 | NM_018390 | Phosphatidylinositol-specific phospholipase C, X domain containing 1 | 1,6 |  | 1 | 2 |
| 53 | CITED2 | NM_006079 | Cbp/p300-interacting transactivator 2 | 1,6 |  |  | 4 |
| 54 | SLC35A1 | NM_006416 | Solute carrier family 35, member A1 | 1,6 |  | 1 | 1 |
| 55 | PMM2 | NM_000303 | Phosphomannomutase 2 | 1,6 |  |  | 1 |
| 56 | SNRPC | NM_003093 | Small nuclear ribonucleoprotein polypeptide C | 1,6 |  |  |  |
| 57 | PPM1B | NM_177968 | Protein phosphatase 1B (formerly 2C), magnesium-dependent, beta isoform | 1,6 |  |  | 1 |
| 58 | GNB1 | NM_002074 | Guanine nucleotide binding protein (G protein), beta polypeptide 1 | 1,6 |  |  | 2 |
| 59 | DDAH1 | NM_012137 | Dimethylarginine dimethylaminohydrolase 1 | 1,6 |  |  | 2 |
| 60 | ARID3A | NM_005224 | AT rich interactive domain 3A | 1,7 |  | 1 |  |
| 61 | LOC285636 | NM_175921 | Hypothetical protein LOC285636 | 1,7 |  | 1 | 2 |

(**C**) 48h after transfection with Pre-miR-17-5p

| 1.5 fold-change | | | | | Number of seed regions | | |
| --- | --- | --- | --- | --- | --- | --- | --- |
| Number | Gene symbol^a^ | Gene accession number^b^ | Gene name | Fold-change^c^ | 5'UTR | CDS | 3'UTR |
| 1 | IL6 | NM_000600 | Interleukin 6 (interferon, beta 2) | -3,4 |  |  |  |
| 2 | KRT17 | NM_000422 | Keratin 17 | -2,1 |  |  |  |
| 3 | C1QTNF1 | NM_198594 | C1q and tumor necrosis factor related protein 1 | -2,0 | 1 |  |  |
| 4 | C8orf4 | NM_020130 | Chromosome 8 open reading frame 4 | -2,0 |  |  | 1 |
| 5 | CYR61 | NM_001554 | Cysteine-rich, angiogenic inducer, 61 | -1,9 |  |  |  |
| 6 | S100P | NM_005980 | S100 calcium binding protein P | -1,9 |  |  |  |
| 7 | CCL20 | NM_004591 | Chemokine (C-C motif) ligand 20 | -1,9 |  |  |  |
| 8 | IRAK2 | NM_001570 | Interleukin-1 receptor-associated kinase 2 | -1,8 |  |  | 1 |
| 9 | SLC16A6 | NM_004694 | Solute carrier family 16, member 6 | -1,8 |  |  | 2 |
| 10 | HSF2BP | NM_007031 | Heat shock transcription factor 2 binding protein | -1,8 |  | 1 |  |
| 11 | TMEM16B | NM_020373 | Transmembrane protein 16B | -1,7 |  |  |  |
| 12 | MMP12 | NM_002426 | Matrix metallopeptidase 12 | -1,7 |  |  |  |
| 13 | WDR69 | NM_178821 | WD repeat domain 69 | -1,7 | 1 |  |  |
| 14 | BIRC3 | NM_001165 | Baculoviral IAP repeat-containing 3 | -1,7 |  | 2 |  |
| 15 | TESC | NM_017899 | Tescalcin | -1,7 |  |  | 1 |
| 16 | IL32 | NM_001012633 | Interleukin 32 | -1,6 |  |  |  |
| 17 | CA9 | NM_001216 | Carbonic anhydrase IX | -1,6 |  |  |  |
| 18 | LDLR | NM_000527 | Low density lipoprotein receptor | -1,6 |  |  | 5 |
| 19 | WDR54 | NM_032118 | WD repeat domain 54 | -1,6 |  |  | 1 |
| 20 | GDF15 | NM_004864 | Growth differentiation factor 15 | -1,6 |  |  |  |
| 21 | CXCR7 | NM_001047841 | Chemokine (C-X-C motif) receptor 7 | -1,6 |  |  |  |
| 22 | CMKOR1 | NM_020311 | Chemokine orphan receptor 1 | -1,6 |  |  |  |
| 23 | AOX1 | NM_001159 | Aldehyde oxidase 1 | -1,6 |  |  |  |
| 24 | RNH1 | NM_203385 | Ribonuclease/angiogenin inhibitor 1 | -1,6 |  |  | 2 |
| 25 | NKD2 | NM_033120 | Naked cuticle homolog 2 | -1,6 |  |  |  |
| 26 | RGS2 | NM_002923 | Regulator of G-protein signalling 2 | -1,5 |  |  |  |
| 27 | SRGN | NM_002727 | Serglycin | -1,5 |  | 1 | 1 |
| 28 | LOC205251 | NM_174925 | Hypothetical protein LOC205251 | -1,5 |  |  |  |
| 29 | MUC1 | NM_001018021 | Mucin 1, transmembrane | -1,5 |  |  |  |
| 30 | FYCO1 | NM_024513 | FYVE and coiled-coil domain containing 1 | -1,5 |  |  | 5 |
| 31 | SERPINB8 | NM_002640 | Serpin peptidase inhibitor, clade B (ovalbumin) | -1,5 | 1 | 2 | 2 |
| 32 | ADAM9 | NM_003816 | ADAM metallopeptidase domain 9 | -1,5 |  |  | 1 |
| 33 | C21orf25 | NM_199050 | Chromosome 21 open reading frame 25 | -1,5 |  |  | 6 |
| 34 | C1S | NM_001734 | Complement component 1, s subcomponent | -1,5 | 1 | 2 |  |
| 35 | DNAJC12 | NM_201262 | DnaJ (Hsp40) homolog, subfamily C, member 12 | -1,5 |  |  |  |
| 36 | TMPRSS3 | NM_032405 | Transmembrane protease, serine 3 | -1,5 | 1 |  |  |
| 37 | PITPNC1 | NM_181671 | Phosphatidylinositol transfer protein, cytoplasmic 1 | -1,5 |  |  | 1 |
| 38 | C9orf152 | NM_001012993 | Chromosome 9 open reading frame 152 | -1,5 |  |  |  |
| 39 | TBC1D2 | NM_018421 | TBC1 domain family, member 2 | -1,5 |  |  | 1 |
| 40 | STEAP4 | NM_024636 | STEAP family member 4 | 1,5 |  |  | 4 |
| 41 | SCNN1A | NM_001038 | Sodium channel, nonvoltage-gated 1 alpha | 1,5 | 1 | 1 |  |
| 42 | CRIP1 | NM_001311 | Cysteine-rich protein 1 | 1,5 |  | 1 |  |
| 43 | GPR37 | NM_005302 | G protein-coupled receptor 37 | 1,5 |  | 2 | 1 |
| 44 | TST | NM_003312 | Thiosulfate sulfurtransferase | 1,5 |  |  |  |
| 45 | RPESP | NM_153225 | RPE-spondin | 1,5 |  |  | 1 |
| 46 | VAV3 | NM_006113 | Vav 3 oncogene | 1,5 |  |  | 3 |
| 47 | PPFIBP2 | NM_003621 | PTPRF interacting protein, binding protein 2 | 1,5 |  | 1 | 1 |
| 48 | SLC12A3 | NM_000339 | Solute carrier family 12 (sodium/chloride transporters), member 3 | 1,6 |  | 2 | 2 |
| 49 | SNRPC | NM_003093 | Small nuclear ribonucleoprotein polypeptide C | 1,6 |  |  |  |
| 50 | SUSD2 | NM_019601 | Sushi domain containing 2 | 1,6 |  | 2 |  |
| 51 | FGFR3 | NM_000142 | Fibroblast growth factor receptor 3 | 1,6 |  |  |  |
| 52 | KRT8 | NM_002273 | Keratin 8 | 1,6 |  |  |  |
| 53 | TNFSF9 | NM_003811 | Tumor necrosis factor (ligand) superfamily, member 9 | 1,6 |  |  |  |
| 54 | MGP | NM_000900 | Matrix Gla protein | 1,6 |  |  |  |
| 55 | ENO3 | NM_001976 | Eolase 3, beta | 1,6 |  |  |  |
| 56 | SNCG | NM_003087 | Synuclein, gamma | 1,6 |  |  |  |
| 57 | LOC91461 | NM_138370 | Hypothetical protein BC007901 | 1,6 |  |  |  |
| 58 | ZNF467 | NM_207336 | Znc finger protein 467 | 1,6 |  |  |  |
| 59 | S100A4 | NM_002961 | S100 calcium binding protein A4 | 1,7 |  |  | 1 |
| 60 | CRYAB | NM_001885 | Systallin, alpha B | 1,7 |  |  |  |
| 61 | SPINK4 | NM_014471 | Srine peptidase inhibitor, Kazal type 4 | 1,7 |  |  |  |
| 62 | ALDH1A3 | NM_000693 | Aldehyde dehydrogenase 1 family, member A3 | 1,8 |  |  | 1 |
| 63 | CLCA2 | NM_006536 | Chloride channel, calcium activated, family member 2 | 1,9 |  |  | 1 |
| 64 | SLC1A3 | NM_004172 | Solute carrier family 1, member 3 | 1,9 |  | 1 | 2 |
| 65 | OLFML1 | NM_198474 | Olfactomedin-like 1 | 1,9 |  | 1 |  |
| 66 | KRTHB1 | NM_002281 | Keratin 81 | 1,9 |  |  | 1 |
| 67 | KRT86 | NM_002284 | Keratin 86 | 1,9 |  |  |  |
| 68 | ACTA2 | NM_001613 | Actin, alpha 2 | 2,1 | 1 |  | 1 |
| 69 | TAC3 | NM_001006667 | Tachykinin 3 | 3,7 |  |  |  |

^a^Official gene symbol in NCBI database

^b^GeneBank gene accession number

^c^mRNA level change in linear range
